# Large‐Scale Synthesis of Multifunctional Single‐Phase Co_2_C Nanomaterials

**DOI:** 10.1002/advs.202301073

**Published:** 2023-04-24

**Authors:** Zhengyi Yang, Tingting Zhao, Shuyan Hao, Rutao Wang, Chunyan Zhu, Yunxiang Tang, Chan Guo, Jiurong Liu, Xiaodong Wen, Fenglong Wang

**Affiliations:** ^1^ Key Laboratory for Liquid–Solid Structural Evolution and Processing of Materials Ministry of Education Shandong University Jinan 250061 P. R. China; ^2^ State Key Laboratory of Coal Conversion Institute of Coal Chemistry Chinese Academy of Sciences Taiyuan 030001 P. R. China; ^3^ National Energy Center for Coal to Liquids Synfuels China Co. Ltd. Huairou District Beijing 101400 P. R. China; ^4^ Beijing Advanced Innovation Center for Materials Genome Engineering Beijing Information S & T University Beijing 101400 P. R. China; ^5^ Shenzhen Research Institute of Shandong University Shenzhen 518057 P. R. China

**Keywords:** cobalt carbide, CO_2_ conversion, large‐scale synthesis, multi‐functional nanomaterials, Pt‐group–like electronic properties

## Abstract

Achieving scalable synthesis of nanoscale transition‐metal carbides (TMCs), regarded as substitutes for platinum‐group noble metals, remains an ongoing challenge. Herein, a 100‐g scale synthesis of single‐phased cobalt carbide (Co_2_C) through carburization of Co‐based Prussian Blue Analog (Co‐PBA) is reported in CO_2_/H_2_ atmosphere under mild conditions (230 °C, ambient pressure). Textural property investigations indicate a successful preparation of orthorhombic‐phased Co_2_C nanomaterials with Pt‐group–like electronic properties. As a demonstration, Co_2_C achieves landmark photo‐assisted thermal catalytic CO_2_ conversion rates with photo‐switched product selectivity, which far exceeds the representative Pt‐group‐metal–based catalysts. This impressive result is attributed to the excellent activation of reactants, colorific light absorption, and photo‐to‐thermal conversion capacities. In addition to CO_2_ hydrogenation, the versatile Co_2_C materials show huge prospects in antibacterial therapy, interfacial water evaporation, electrochemical hydrogen evolution reaction, and battery technologies. This study paves the way toward unlocking the potential of multi‐functional Co_2_C nanomaterials.

## Graphical abstract

A 100‐g scale synthesis of single‐phased cobalt carbide (Co_2_C) through carburization of Co‐based Prussian Blue Analog (Co‐PBA) in CO_2_/H_2_ atmosphere under mild conditions is reported. The orthorhombic Co_2_C exhibits Pt‐group–like electronic structure. Impressively, the Co_2_C materials show huge prospects in photo‐assisted thermal catalytic CO_2_ conversion, antibacterial therapy, interfacial water evaporation, hydrogen evolution reaction, and battery technologies.

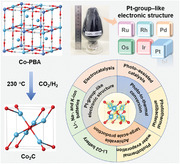

## 1 Introduction

Transition‐metal carbides (TMCs), as members of interstitial alloys, are vividly considered by inserting carbon atoms orderly into the metal lattices.^[^ ^1^ ^]^ As the pioneering work on tungsten carbide with platinum‐like catalytic behaviors,^[^ ^2^ ^]^ it became evident that the electronic features of TMCs can be modulated by hybridization between the alloying carbon s‐ and p‐ orbitals with the d‐ orbitals of the host metal; thus, imparting surface properties similar to that of platinum and other precious metals.^[^ ^3^ ^]^ As such, common TMCs, including Mo_2_C, Ti_3_C_2_ (MXenes), Nb_3_C_2_, VC, WC, and Fe_5_C_2_, have attracted ever‐growing attention for various applications owing to their novel intrinsic characteristics.^[^ ^4^ ^]^

Cobalt carbide (Co_2_C), a prospective alternative to platinum‐group metals, has been intensively reported for catalytic applications, especially in the Fischer–Tropsch synthesis (FTS).^[^ ^5^ ^]^ Initially, the formation of Co_2_C was considered as an indicator for the deactivation of cobalt‐based catalysts during FTS until the work of the Sun group was reported, in which the authors found that Co_2_C, as the active sites of CoMn catalysts, could efficiently promote the production of lower olefins from syngas.^[^ ^6^ ^]^ In addition, Co_2_C also exhibited a promising catalytic activity in reverse water gas shift (RWGS), hydrogen evolution reaction, and CO_2_–epoxide cycloaddition reaction.^[^ ^7^ ^]^ Up to now, Co_2_C could be obtained through the following routes: i) high‐temperature wet‐chemistry approaches (>300 °C) through the pyrolysis of cobalt carbonyl compounds in organic alcohol/amine agents under inert gas atmosphere,^[^ ^7b,c^ ^]^ ii) temperature‐programmed carburization processes via the FTS reaction using syngas as carbon source,^[^ ^3^, ^5^, ^6^ ^]^ iii) laser ablation methods using metallic cobalt and carbon‐rich precursor;^[^ ^8^ ^]^ iv) physical vapor deposition (PVD) method using Co–C composite targets,^[^ ^9^ ^]^ and v) H_2_ plasma‐assisted atomic layer deposition (ALD).^[^ ^10^ ^]^ Nevertheless, these sophisticated techniques suffer from the shortcomings of harsh experimental conditions, by‐product formation, and especially the difficulty in scalable production, which impedes the efforts to unlock their fundamental properties and reach multiple application potential. As such, it is highly desirable to establish new synthetic methodology for effortless cobalt carbide production to explore the prospects of these materials for noble‐metal substitution and other applications.

In this study, we report a large‐scale synthetic strategy for the production of single‐phase Co_2_C nanoparticles with great industrial perspectives, which is achieved through carburization of Co‐based Prussian Blue Analog (Co‐PBA) carburization in a CO_2_/H_2_ atmosphere under mild conditions (230 °C, ambient pressure), and which is, to the best of our knowledge, the first report of industrial‐scale synthesis of Co_2_C. The produced orthorhombic‐phased Co_2_C nanomaterials with Pt‐group–like electronic structure show excellent light absorption capacity covering the UV–vis–IR region (300–2500 nm). Subsequently, we used photo‐assisted thermal catalytic CO_2_ hydrogenation as a probe reaction to investigate the performance of the obtained Co_2_C nanomaterials for noble‐metals substitution. The results indicated that Co_2_C could provide an exceptional catalytic activity for the hydrogenation of CO_2_ with H_2_ to form hydrocarbons (C_1_), and the product selectivity could be switched from CO to CH_4_ by a subsequent photo‐activated *CO hydrogenation process. Attributed to the remarkable activation of reactants, strong light absorption, and photo‐to‐thermal conversion ability, Co_2_C catalysts achieved, with the assistance of 1900 W m^−2^ light irradiation, a CO_2_ conversion rate of 123.0 mmol g_cat_^−1^ h^−1^ with CH_4_ selectivity of 80%. In the absence of light irradiation, our catalyst achieved a 65.3 mmol g_cat_^−1^ h^−1^ conversion rate with CO selectivity of 85%. In both cases, the reactor temperature was set to 300 °C. These superior activities far exceeded the referenced Pt‐group‐metal–based catalysts (Pt/Al_2_O_3_ and Ru/Al_2_O_3_), establishing a new milestone in CO_2_ hydrogenation. Furthermore, the prepared Co_2_C materials also exhibited intriguing application potential in photothermal/photodynamic antibacterial therapy, interfacial steam generation, electrocatalytic hydrogen evolution reaction, Li‐, Na‐, and K‐ion battery anodes, and Li–O_2_ battery cathode materials. The findings in this work will almost certainly lay the foundations for exploring the multiple functions of cobalt carbide in industrial trials.

## 2 Results and Discussion

**Figure 1:**
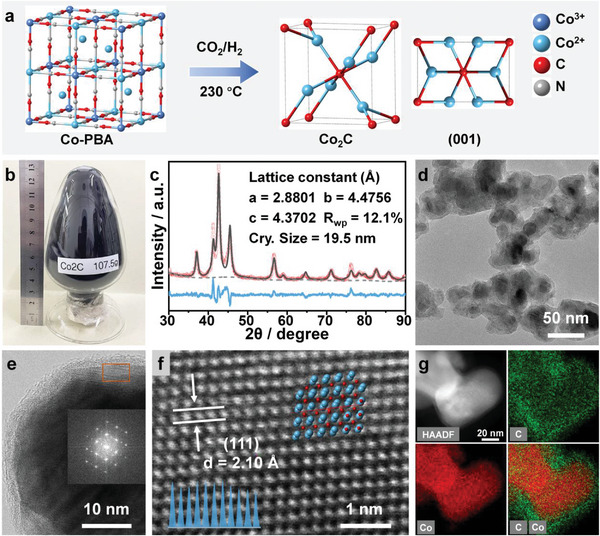
a) Schematic illustration of the preparation procedure for Co_2_C. b) Digital photograph of Co_2_C from one‐batch synthesis with a net weight of 107.5 g. c) Rietveld refinement of XRD pattern of Co_2_C in the range of 30–90°. Raw data, fitting profile, differences, and background are shown as red open circles, black lines, light blue lines, and grey lines, respectively. d) HR‐TEM and e) HAADF‐STEM images of Co_2_C. The inset shows the corresponding SAED patterns. f) The magnified image of rectangular region in (e). g) The corresponding EDX elemental mappings.

As illustrated in **Figure** **1**a, nanoscale Co_2_C is fabricated through one‐step carburizing process of Co‐PBA in CO_2_/H_2_ gas mixture. Figure 1b; Figures S1 and S2, showing batch synthesis of Co_2_C and explorations of synthetic conditions, indicate the tremendous industrial potential of the above method for metal carbides production, which is achievable at mild synthetic conditions (230 °C or 170 °C with light irradiation, ambient pressure), performed using straightforward procedures and features tunable metal coordinated frameworks. Online gas chromatography (GC) confirms that the Co_2_C, formed in CO_2_/H_2_ stream, could in situ catalyze CO_2_ hydrogenation to hydrocarbons (Figure S3, Supporting Information). In addition, the in situ generated CO was proven to promote the formation of Co_2_C in turn (Figure S4, Supporting Information). Carbon‐13 isotope tracing experiments further reveal that the carbon element in Co_2_C initially originated from both ^13^CO_2_ and Co‐PBA (^12^C) (Figure S5, Supporting Information). As carburization progressed, the Co_2_^12^C would be converted to Co_2_^13^C, gradually attributed to dynamic chemical equilibrium (Figures S6 and S7, Supporting Information). Thus, the involved carburization (Equation 1) and catalytic processes (Equations (2) and (3) accounting for the Co_2_C formation could be plausibly proposed as below (Figure S8, Supporting Information):

(1) $$\begin{array}{ccc} {}^{13}{\mathrm{CO}}_{2} & + & H_{2}+\mathrm{Co}-\mathrm{PBA}\to {\mathrm{Co}}_{2}^{12/13}C{+}^{12/13}C_{1}{+}^{12/13}{\mathrm{CO}}_{2}\\ & + & \left({\mathrm{NH}}_{4}\right){\mathrm{CO}}_{2}\left(\mathrm{OH}\right) \end{array}$$

(2) $${}^{13}{\mathrm{CO}}_{2}+H_{2}+Co_{2}^{12}C\to {\mathrm{Co}}_{2}^{13}C{+}^{12/13}C_{1}$$

(3) $${}^{13}{\mathrm{CO}}_{2}+H_{2}+Co_{2}^{13}C\to {\mathrm{Co}}_{2}^{13}C{+}^{13}C_{1}$$

where ^12/13^C_1_ represents both ^12^C and ^13^C labeled hydrogenation products of CO_2_, including CO, CH_4,_ and CH_3_OH.

The Rietveld refinement of X‐ray diffraction (XRD) pattern indicates that Co_2_C adopts an orthorhombic structure (Pmnn) with an estimated crystal size of 19.5 nm (Figure 1c). This crystal structure can be described as one in which the Co atoms assemble in a *hcp* arrangement, while the carbon atoms fill the octahedral interstices by forming a *bcc* crystal structure (Figure S9, Supporting Information). The high‐resolution transmission electron microscopy (HR‐TEM) images show that the spherical Co_2_C nanoparticles with sizes of 10–30 nm are encapsulated by a 1–2 nm thin loose layer of carbon atoms that resemble a shell structure, which is regarded as a representative morphological characteristic of metal carbides that provides robust protection against agglomeration and oxidation (Figure 1d,e).^[^ ^5^, ^6^ ^]^ The pattern of selected area electron diffraction (SAED) further elaborates the crystalline structure of Co_2_C (inset of Figure 1e). The high‐angle annular dark field scanning transmission electron microscopy (HAADF‐STEM) images reveal the regular arrangement of constituent atoms (Figure 1f; Figure S10, Supporting Information). Typical Co_2_C(111) planes with characteristic lattice spacing of 2.10 Å are observed, which agrees well with the XRD analysis. The energy‐dispersive X‐ray spectroscopy (EDX) mappings corroborate the homogeneous distribution of C and Co elements in the nanoparticles (Figure 1g).

**Figure 2:**
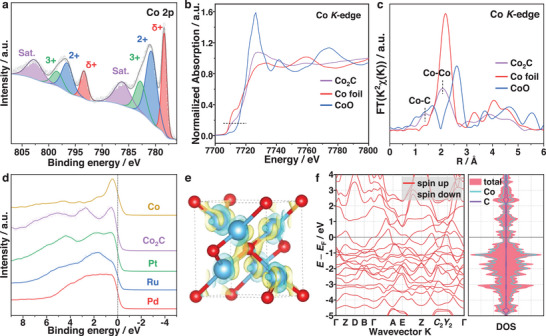
a) Co 2p XPS spectrum of Co_2_C. b) Normalized XANES spectra of Co_2_C, Co foil, and CoO at the Co K‐edge and c) corresponding k^2^‐weighted FT‐EXAFS spectra. d) VBSs of Co_2_C, referenced Co, Pt, Ru, and Pd powder. e) Difference in charge density of Co_2_C model. f) Calculated band structure, partial, and total densities of states.

The electronic structure associated with the chemical properties of Co_2_C nanoparticles is characterized by X‐ray/ultraviolet photoelectron spectroscopy (XPS/UPS) and X‐ray absorption spectroscopy (XAS). As shown in **Figure** **2**a; Figure S11, Supporting Information, four peaks are resolved in Co 2p_3/2_ XPS spectrum. Besides the satellite peak at 786.2 eV, the characteristic peak at 778.3 eV can be assigned to the carbonic Co,^[^ ^7b^ ^]^ which can also be confirmed by the peak of Co—C at 283.3 eV in the C 1s XPS spectrum (Figure S12, Supporting Information). Furthermore, the peaks at 780.8 and 782.8 eV are associated with the oxidized cobalt on the top surface, which agrees with the signal of cobalt oxide species at 529.5 eV in the O 1s XPS spectrum (Figure S13, Supporting Information). Besides, the dominant C—O state of O 1s XPS spectra at 531.7 eV can be indexed to carbonates, revealing the excellent oxidation resistance of cobalt carbide bestowed by surface carbon layers. Co K‐edge X‐ray absorption near‐edge structure (XANES) spectra in Figure 2b indicates that the dominant valence state of Co element in bulk Co_2_C, correlated directly with the position of edge features, is between the reference metallic Co foil and CoO oxides. Each edge position shifts to higher energy regions as the oxidation state increases and the position could be reflected by the inflection point. As a function of energy shift, the fitted Co average valence state of Co_2_C is estimated to be +0.66, which agrees well with the Co 2p_3/2_ XPS spectra (Figures S10 and S14, Supporting Information).^[^ ^11^ ^]^ Figure 2c shows the Fourier transform spectra of extended X‐ray absorption fine structure (EXAFS). The peaks at 1.4 and 2.1 Å are attributed to the scattering paths of Co—C and Co—Co bonds in Co_2_C, respectively. IN addition, wavelet transform (WT)‐EXAFS analysis also indicates that the chemical states and coordination environments of Co atoms are affected by both Co—C and Co—Co species (Figure S15, Supporting Information). Ascribed to the fact that C atoms occupied the octahedral interstices surrounded by Co atoms, structural parameters of EXAFS fitting results show that the Co—C shell has a distance of 1.9 Å with a coordination number of 2.4, which is consistent with previous studies (Figure S16 and Table S1, Supporting Information).^[^ ^12^ ^]^ It may also be deduced that the coordination number of Co—Co shell over Co_2_C is smaller than that in Co foil (3.7 vs 12.0) even though the Co—Co atomic distance remains almost unchanged (2.46 vs 2.49). Apparently, the electronic properties of cobalt carbide, depending on the atomic structural configuration, are regulated with the carburization. Importantly, diffuse reflectance spectra (DRS) in Figure S17, Supporting Information, reveals that Co_2_C, as a saturated light absorber (300–2500 nm), possesses a negligible band gap near the Fermi level, indicating a strong photo‐energy utilization ability. UPS spectrum measurements indicate that the work function (*Φ*) of Co_2_C is 4.84 eV, which is similar to those of the Group‐VIII metals (Figure S18, Supporting Information). Furthermore, inspection of the valence band spectrum (VBS) in Figure 2d qualitatively suggests that the metallic Co_2_C possesses a Pt‐group–like electronic structure, especially that of Pt metal. Owing to the superparamagnetic behavior of Co_2_C without saturation magnetization, as shown in Figure S19, Supporting Information, nonmagnetic density functional theory (DFT) calculations were performed to obtain an in‐depth understanding of the metallic electronic structure of the carbide. The different charge density in Figure 2e confirms that there exists electron transfer from the positively charged Co atoms to the negatively charged C atoms, which is in line with the difference in electronegativity between Co and C elements. Furthermore, the electronic cloud mostly accumulates between Co and C, delineating their strong bonding configuration.^[^ ^13^ ^]^ The calculated band structure, as shown in Figure 2f, reveals that Co_2_C does not exhibit a band gap, which is consistent with the DRS and VBS results. The states around the Fermi level are primarily modified by Co, suggesting the dominance of Co in the electronic structure of Co_2_C.

**Figure 3:**
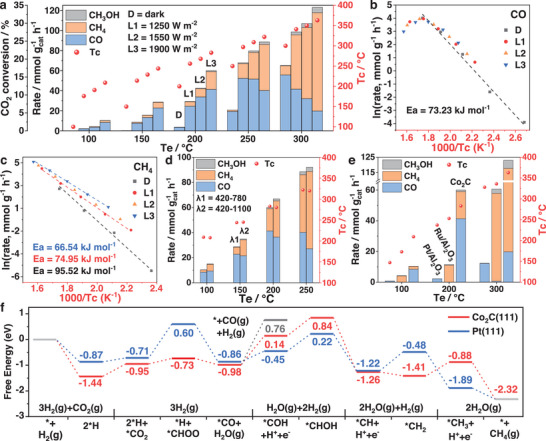
a) Photo‐assisted thermal catalytic CO_2_ hydrogenation performances over Co_2_C as functions of set reactor temperature and light intensity. b) Arrhenius plots for RWGS reaction. c) Arrhenius plots for methanation reaction. d) Production rates as functions of *T*_e_ and irradiation source. e) Production rates over different catalysts under photo‐assisted thermal conditions. f) Free‐energy changes of the Pt(111) and Co_2_C(111) models for catalyzing the CO_2_ hydrogenation to CH_4_.

To demonstrate the potential of Co_2_C for noble‐metals substitution, we evaluated the catalytic performance of the as‐prepared Co_2_C together with Pt/Al_2_O_3_ and Ru/Al_2_O_3_ as representative Pt‐group metal‐based catalysts for photo‐assisted thermal CO_2_ hydrogenation in a flow reactor under light illumination at atmospheric pressure (Figure S20, Supporting Information; Experimental Section). As depicted in **Figure** **3**a, Co_2_C catalysts exhibited outstanding CO_2_ hydrogenation performance. Both the CO_2_ conversion and catalyst temperature (*T*_c_) increased with the rise in external heat temperature (set temperature of reactor, *T*_e_) and light intensity. At *T*_e_ = 250 °C, the CO_2_ conversion rate reached 88.6 and 20.8 mmol g_cat_^−1^ h^−1^ with and without 1900 W m^−2^ (L3) light irradiation, respectively, exhibiting a 326% photo‐enhancement. As the light intensity was decreased to 1250 W m^−2^ (L1), the CO_2_ conversion rate declined to 67.8 mmol g_cat_^−1^ h^−1^, corresponding to a 226% photo‐enhancement. At *T*_e_ = 300 °C, the CO_2_ conversion rate reached 123.0 and 65.3 mmol g_cat_^−1^ h^−1^ with and without L3 light irradiation, respectively, with a photo‐enhancement of 88%. These results indicate that raising *T*_e_ and reducing light intensity is not conducive to the photo‐enhancement. Basically, this photo‐enhancement was considered as comprising simply of photochemical and photothermal enhancements. At *T*_e_ = 250 °C with L1 and L3 irradiation, the *T*_c_ reached 297 °C and 322 °C with ∆*T* (*T*_c_−*T*_e_) of 47 °C and 72 °C, respectively, suggesting a significant photothermal enhancement during the catalytic reaction under light irradiation. Notably, as *T*_e_ was increased, ∆*T* was gradually weakened. Furthermore, at *T*_c_ = 297 °C (*T*_e_ = 250 °C with L1 illumination), the CO_2_ conversion rate reached 67.8 mmol g_cat_^−1^ h^−1^. Concerning the CO_2_ conversion of 65.3 mmol g_cat_^−1^ h^−1^ at nearly 300 °C in the dark, only a 4% photochemical enhancement was observed, meaning that the photo‐enhancement may be regarded as a predominantly photothermal enhanced process. Similar to the photo‐enhancement, increasing the light intensity was also beneficial to the photochemical enhancement and an ≈38% photochemical enhancement was attained when comparing the CO_2_ conversion rate at 250 °C (*T*_e_) in the dark and 150 °C (*T*_e_) under L3 illumination (*T*_c_ = 244 °C).

With the increase in light intensity, and in addition to the high CO_2_ conversion rate, the main product was gradually changed from CO to CH_4_, reflecting the widely tunable product selectivity of Co_2_C catalyst. In the dark, the high CO selectivity (≈85–95%) across the studied temperature range indicated that the RWGS reaction was a thermal‐driven process. Under light illumination, the much‐enhanced CH_4_ production selectivity clearly showed that the formation of CH_4_ was a photo‐activated process. At *T*_e_ = 300 °C, the CH_4_ selectivity reached 80% and 14% (CO selectivity: 85%) with and without L3 irradiation, respectively, further indicating that the products could be switched from CO to CH_4_ under light irradiation. More interestingly, besides the selectivity, the CO production rate was also suppressed under light irradiation at elevated temperatures. On accounts of the endothermic nature of the RWGS reaction, in which raised temperature can markedly accelerate the reaction rate, the actually decreased CO production rate was strongly correlated to the further hydrogenation of the diffused *CO intermediates to CH_4_ owing to the photo‐activated process.

To elucidate the detailed reaction mechanism of CH_4_ formation, the reaction kinetics of RWGS and CH_4_ formation were analyzed. As shown in Figure 3b and as deduced from linear Arrhenius plots, in the dark, the apparent activation energy (*E*_a_) for CO formation was 73.23 kJ mol^−1^. Under light irradiation, the almost unchanged *E*_a_ at low temperatures (*T*_c_ < 280 °C) implied a similar reaction mechanism to that in the dark, and the light energy was mainly converted into thermal energy to promote the reaction. Nevertheless, at higher temperatures, the parabolic Arrhenius plots for CO production indicated violent side chemical reactions in which adsorbed CO species (*CO) acted as reactants. Given the experimentally dominant CH_4_ production in CO hydrogenation reactions (Figure S21, Supporting Information) and the linear Arrhenius plots for CH_4_ formation (Figure 3c), we can conclude that the CH_4_ was produced through *CO hydrogenation pathway, and Co_2_C worked as a photo‐switched dual‐function catalyst during the photo‐assisted thermal CO_2_ hydrogenation process (Figure S22, Supporting Information). Different from the RWGS reaction, under L1 light irradiation, the *E*_a_ of CH_4_ formation declined to 74.95 kJ mol^−1^. The decreased *E*_a_ for CH_4_ formation under light irradiation further indicated that the CH_4_ formation was a photo‐activated process. Furthermore, this activation was more apparent when increasing the light intensity and extending the light spectrum region (Figure 3c,d).

As shown in Figure 3e, the main products of Pt/Al_2_O_3_ and Ru/Al_2_O_3_ catalyst were CO and CH_4_, respectively. Compared with Pt/Al_2_O_3_ and Ru/Al_2_O_3_ catalysts, and with the same input energy during the reaction (same *T*_e_), Co_2_C catalyst exhibited drastically enhanced catalytic activity and photo‐to‐thermal conversion ability (characterized by the ∆*T*). At *T*_e_ = 300 °C under L3 irradiation, the CO_2_ conversion efficiency of Co_2_C was ≈10 and 2 times higher than those of Pt/Al_2_O_3_ and Ru/Al_2_O_3_ catalysts, respectively. In particular, the CO production rate was 1.6 times higher than that of the Pt/Al_2_O_3_ catalyst, and the CH_4_ production rate was 1.7 times higher than that of the Ru/Al_2_O_3_ catalyst. To the best of our knowledge, Co_2_C catalyst achieved a landmark CO_2_ hydrogenation performance that far exceeded those of the commonly used noble‐metal–based catalysts reported to date (Table S2, Supporting Information). A 25‐h continuous test at *T*_e_ = 300 °C under L3 illumination indicated that Co_2_C could efficiently and durably catalyze CO_2_ hydrogenation for methane production (Figure S23, Supporting Information).

To unlock the electronic origin of the promising catalytic performance of the Co_2_C catalyst, first‐principles calculations were conducted to investigate the CO_2_ methanation reaction mechanisms. Based on the experimental results, we constructed two catalyst models, one for Co_2_C(111) and another for Pt(111), to simulate the Co_2_C and Pt‐group‐metal catalysts, respectively. The calculated RWGS reaction followed the formate (*HCOO) pathway, and the subsequent methanation reaction followed the methoxy (*COH) pathway, which agreed with the results of in situ diffuse reflectance infrared Fourier transform spectroscopy (in situ DRIFTS) measurements in Figure S24, Supporting Information. As shown in Figure 3f; Figure S25, Supporting Information, both the adsorption of CO_2_ (*CO_2_) and the formation of *HCOO, *COH, and hydroxymethylene (*CHOH) intermediates were endothermic processes. The adsorbed CO was more likely to be further hydrogenated with H to form *COH intermediate (energy change: 1.12 eV) than to be directly desorbed (energy change: 1.74 eV), which is in line with the experimentally elevated CH_4_ selectivity under light irradiation. Notably, the Co_2_C(111) and Pt(111) models indicated similar energy trends during the whole reaction, which might be attributed to their similar electronic properties. DFT calculations indicated that Co_2_C(111) was more favorable for the H_2_ dissociation and adsorbed CO_2_ activation than Pt(111). For the Pt(111) site, the rate‐determining step (RDS) was the hydrogenation of *CO_2_ (−0.71 eV) to *HCOO (0.60 eV) with an energy change of 1.31 eV. For the Co_2_C(111) site, the RDS was the hydrogenation of *CO (−0.98 eV) to *COH (0.14 eV) with an energy change of 1.12 eV. In contrast to Pt(111) active sites, the Co_2_C(111) facets exhibited a higher catalytic ability for the hydrogenation of CO_2_ to CH_4_, which is ascribed to the more robust activation to key intermediates in the reaction process. This finding confirms the huge prospects of using Co_2_C materials as potential catalyst substitutes for Pt‐group noble metals.

**Figure 4:**
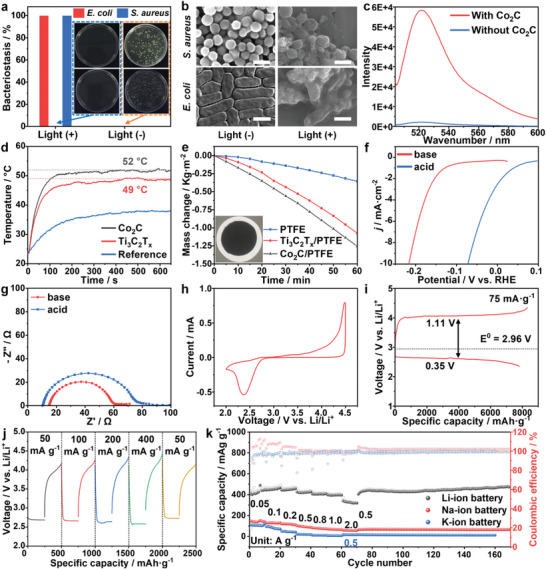
Photothermal/photodynamic antibacterial performances: a) Antibacterial efficiency of Co_2_C against *E. coli* and *S. aureus* under light irradiation. b) SEM images of *E. coli* and *S. aureus* before/after light irradiation for 15 min treated with 200 µg mL^−1^ of Co_2_C. c) Fluorescence emission spectra of Co_2_C with reference H_2_O. Solar‐driven interfacial water evaporation: d) photo‐to‐thermal conversion capacity comparison of Co_2_C materials with referenced MXenes and e) water evaporation performance of Co_2_C/PTFE membrane as a function of irradiation time. Electrochemical hydrogen evolution reaction: f) polarization curves of Co_2_C electrocatalyst in 1 m of KOH and 0.5 m of H_2_SO_4_ electrolytes and g) the corresponding electrochemical impedance spectra. Electrochemical Li—O_2_ battery: h) cyclic voltammetry (CV) curve of Co_2_C cathodes at 0.10 mV s^−1^ within a voltage window of 2.0–4.5 V, i) initial discharge/charge profiles of Co_2_C cathodes at 75 mA g^−1^ from 2.35 to 4.35 V, and j) rate capability of Co_2_C cathodes. Electrochemical Li‐, Na‐, and K‐ion batteries: k) rate capacities and cycling performances of Co_2_C electrode as Li‐, Na‐, and K‐ion anodes at varied current density.

]Encouraged by these results, and inspired by the possibilities of achievable large‐scale production, full spectrum light absorption, and excellent photo‐to‐thermal conversion capacity, we explored multifunctional applications of Co_2_C materials. **Figure** **4**a–c; Figure S26, Supporting Information shows that Co_2_C materials offer great prospects in photothermal/photodynamic antibacterial therapy with high reactive‐oxygen‐species (ROS) generation and a nearly 100% antibacterial efficiency against *Escherichia coli* and *Staphylococcus aureus* under 8 suns (0.8 W cm^−2^) radiation. Compared with the few‐layered MXenes (a group of promising materials for photothermal applications), the Co_2_C powder exhibited a higher photo‐to‐thermal conversion, showing its excellent prospects for photothermal applications (Figure 4d). In line with this, we imbedded Co_2_C nanomaterials into polytetrafluoroethylene (PTFE) membrane and found that under one sun of illumination, the Co_2_C/PTFE composite membrane achieved a water evaporation rate of up to 1.26 kg m^−2^ h^−1^ (Figure 4e; Figure S27, Supporting Information), which exceeded the referenced Ti_3_C_2_T*_x_*/PTFE sample. Furthermore, benefiting from the Pt‐group–like electronic structure, Co_2_C materials also exhibited enormous potential in electrocatalytic processes. As shown in Figure S28, Supporting Information, as electrocatalysts for hydrogen evolution reaction, Co_2_C materials exhibited high electrocatalytic activity with low charge transfer resistance (Figure 4f,g). In addition, under a cathodic current density of 10 mA cm^−2^, the overpotential reached 75 mV with a 0.5‐m H_2_SO_4_ electrolyte and 216 mV with a 1.0‐m KOH electrolyte. Moreover, the synthesized Co_2_C nanomaterials also found applications as electrode materials in batteries. For example, with Li–O_2_ cathode and Li‐, Na‐, or K‐ion battery anode materials, Co_2_C achieved favorable rate performance with good electronic conductivity, cyclic stability, reversibility, and high coulombic efficiency (Figure 4h–k; Figures S29–S32, Supporting Information). In the Li–O_2_ battery, Co_2_C delivered a specific capacity of 7814 mAh g^−1^ at a current density of 75 mA g^−1^ (Figure 4i). In the Li‐, Na‐, and K‐ion batteries, Co_2_C achieved specific capacities of 440.4, 132.9, and 105.4 mAh g^−1^, respectively, at a current density of 0.1 A g^−1^ (Figure 4k). These explorations clearly demonstrate the great application potential of Co_2_C, which could find use in multi‐functional platforms. To that end, we are currently further optimizing its performance through hybridization with other components, adjustment of morphology, doping with other metal ions, and so on.

## 3 Conclusion

We have developed a mild carburization strategy for the synthesis of orthorhombic Co_2_C nanoparticles, which exhibited great industrial perspectives owing to the scalable procedures. Structure characterizations revealed that the Co_2_C materials, possessing saturated light absorption capacity and Pt‐group–like electronic properties, exhibited fascinating catalytic performances. In photo‐assisted thermal catalytic process, Co_2_C, as a single homogeneous catalyst, achieved landmark CO_2_ hydrogenation performances, far exceeding those of the representative Pt‐group‐metal catalysts. Furthermore, the versatile Co_2_C also displayed huge prospects in photothermal therapy, water evaporation, and electrocatalysis for hydrogen evolution, as well as for Li—O_2_ and Li‐, Na‐, and K‐ion batteries. We believe that this work will substantially advance the progress for controllable preparation of metal carbides and their subsequent multi‐application exploitation.

## 4 Experimental Section

### Chemicals and Materials

Cobalt acetate (Co(CH_3_COO)_2_ 4H_2_O, 99.9% metals basis), potassium hexacyanocobaltate (K_3_(Co(CN)_6_, 99%), hydroxydiacetyl iron (Fe(CH_3_COO)_3_ nH_2_O,AR), cobalt (II) oxide (CoO, AR), tricobalt tetrooxide (Co_3_O_4_, AR), cobalt (Co powder, 99.9% metals basis), platinum (Pt powder, 99.9% metals basis), palladium (Pd powder, 99.5% AR), ruthenium (Ru powder, 99.99% metals basis), chloroplatinic acid hexahydrate (H_2_PtCl_6_ 6H_2_O, AR, Pt ≥ 37.5%), ruthenium (III) chloride hydrate (RuCl_3_ *x*H_2_O, 99.99% metals basis), aluminum oxide (Al_2_O_3_, 99.99%), titanium oxide (TiO_2_, 99.8%), few‐layered MXenes (Ti_3_C_2_T*_x_*), polytetrafluoroethylene membrane (PTFE, mouth diameter: 50 mm, pore size: 0.45 µm) porous quartz sands (AR), and quartz wool (2–5 µm) were purchased from Macklin. Carbon dioxide (CO_2_, 99.999%), carbon monoxide (CO, 1.000%), hydrogen (H_2_, 99.999%), oxygen (O_2_, 99.9%), and nitrogen (N_2_, 99.999%) were obtained from Jinan Xusheng Gas Co., Ltd. Carbon‐13 isotope (^13^CO_2_, 99.0 atom%) was purchased from Sigma–Aldrich. All the reagents were used directly without further purification. Deionized water was used for all experiments.

### Synthesis of Catalysts

Nanoscale Co_2_C was fabricated by carburizing Co‐PBA (Co_3_[Co(CN)_6_]_2_) using CO_2_/H_2_ gas through temperature‐programmed reduction method. Typically, 1.125 mol of Co(CH_3_COO)_2_∙4H_2_O was dissolved in 4000 mL ultrapure water, giving the solution A. In parallel, 0.75 mol of K_3_(Co(CN)_6_ was dispersed into 4000 mL ultrapure water, forming the solution B. Then, the solution A was gradually mixed with solution B under stirring. The obtained pink suspension was kept overnight for aging and precipitating. After being washed and dried, the bright purple Co‐PBA precursor was carburized in a tube furnace with a CO_2_/H_2_ mixture (25/100 sccm) flow at 230 °C (or in the photo‐assisted thermal catalytic micro reactor with a CO_2_/H_2_ [4/16 sccm] stream at 170 °C under visible light irradiation) for 10 h to get the final black Co_2_C power. Due to the mild and simple synthetic procedures, the synthesis of Co_2_C could be easily scaled up by increasing the raw chemicals proportionally.

Referenced Pt/Al_2_O_3_ and Ru/Al_2_O_3_ samples were prepared through a wetness impregnation method using H_2_PtCl_6_·6H_2_O and RuCl_3_·*x*H_2_O as metal precursors and NaBH_4_ solution as the reducing agent. Typically, 200 mg of Al_2_O_3_ and metal precursors (metal loading: 1.0 wt%) were dispersed into 100 mL of Mili‐Q water by sonication; and then, excessive NaBH_4_ solution (0.1 mol L^−1^) was quickly added into the suspension under stirring. After washing and drying, the samples were collected for further use.

### Characterizations

The crystal structures of the studied catalysts were characterized by X‐ray diffraction (XRD, DMAX‐2500 PC) using a Cu K*α* source. The micromorphology was recorded using transmission electron microscopy (TEM, JEM‐1011) and high‐resolution STEM (HR‐STEM, Thermo Fisher Spectra 300) with energy‐dispersive X‐ray spectroscopy (EDX, Super X). The C‐13 isotope tracing experiments were performed using gas chromatography–mass spectrometry (GC–MS, Shimadzu TQ8040). The surface chemical states and electronic band structures of the studied materials were identified by X‐ray photoelectron spectroscopy (XPS, AXIS Supra) and ultraviolet photoelectron spectroscopy (UPS, Thermo Scientific, ESCALAB 205), and the data were calibrated with the C1s peak at 284.8 eV. The valence band spectrum (VBS) of the reference metal powder was calibrated with differentiation at 0.0 eV. XAS analysis of the Co K‐edge was conducted at the Singapore and Shanghai Synchrotron Radiation Facilities, and standard Co foil and CoO were employed as the reference samples. The optical properties of the samples were determined using a UV–vis–NIR spectrophotometer (DRS; Hitachi U‐4100). Magnetic behavior was recorded using vibrating sample magnetometry (VSM, LakeShore 7404). The light intensity of a 300‐W Xe lamp (CEL‐HXF300‐T3, 190–1100 nm) was controlled by adjusting the current and calibrated using an automatic strong light power meter (CEL‐NP2000).

### Catalytic Performance Evaluation

As shown in Figure S20, Supporting Information, the CO_2_ hydrogenation reaction over the Co_2_C catalysts was investigated in a miniature photo‐assisted thermal catalytic micro‐reaction system (Beijing China Education Au‐light Co., China) with a quartz window to allow light to penetrate. The quartz flow reactor was placed in a combined external heat furnace. The irradiation was 5.76 cm^2^ (24 mm × 24 mm), and the inner width of the quartz flow reactor was 2.0 mm. Typically, 30 mg of the catalyst was mixed with 1.25 g of porous quartz sand, and the mixture was packed in a quartz reactor. The mixture was fixed using quartz wool, which was placed at both ends of the quartz reactor. The real temperature of the catalysts was measured using a thermocouple with a diameter of 1.5 mm, the tip of which was inserted into the catalyst layer (Figure S20a–c, Supporting Information). A 1:4 mixture of CO_2_ and H_2_ at 0.1 MPa passed through the reactor at a flow rate of 20 mL min^−1^. The gas components from the outlet were analyzed using a gas chromatograph (GC7920) equipped with a flame ionization detector (FID1 and FID2) and a thermal conductivity detector (TCD) using N_2_ as the carrier gas. In this study, the products were CH_4_, CO, and CH_3_OH.

For photo‐assisted thermal CO_2_ hydrogenation, the catalysts were irradiated with a 300‐W Xe lamp equipped with a visible light filter (420–780 nm), and the heat was provided by an external furnace (Figure S20d,e, Supporting Information).

In all tests, light irradiation significantly enhanced the catalytic behavior. Generally, photo‐enhancement comprises of photochemical and photothermal enhancements. In this work, photo‐enhancement and photochemical enhancement were evaluated by comparing the CO_2_ conversion efficiency at the same Te and Tc with and without light irradiation, respectively. The CO_2_ conversion rate (mmol g_cat_^−1^ h^−1^), CO production rate (mmol g_cat_^−1^ h^−1^), CH_4_ production rate (mmol g_cat_^−1^ h^−1^), and product selectivity (%) were calculated using the following equations:

(4) $$C_{\mathrm{CO}2}=\frac{A_{\mathrm{CO}2,\mathrm{in}}-\;A_{\mathrm{CO}2,\mathrm{out}}}{A_{\mathrm{CO}2,\mathrm{in}}}$$

(5) $${\mathrm{CO}}_{2}\mathrm{conversion}\;\;\mathrm{rate}=\frac{C_{\mathrm{CO}2}\times v_{\mathrm{CO}2}\times h}{V_{M}\times m}$$

(6) $$S_{\mathrm{CO}}=\frac{A_{\mathrm{CO}}}{A_{\mathrm{CO}}+A_{\mathrm{CH}4}+A_{\mathrm{CH}3\mathrm{OH}}}$$

(7) $$\mathrm{CO}\;\;\mathrm{production}\;\;\mathrm{rate}={\mathrm{CO}}_{2}\mathrm{conversion}\;\;\mathrm{rate}\times S_{\mathrm{CO}}$$

(8) $${\mathrm{CH}}_{4}\mathrm{production}\;\;\mathrm{rate}={\mathrm{CO}}_{2}\mathrm{conversion}\;\;\mathrm{rate}\times S_{\mathrm{CH}4}$$

(9) $$\mathrm{Photo}-\mathrm{enhancement}=\frac{C_{\mathrm{CO}2,T_{e}+\mathrm{light}}-\;C_{\mathrm{CO}2,T_{e}\;\;\mathrm{in}\;\;\mathrm{dark}}}{C_{\mathrm{CO}2,T_{e}\;\;\mathrm{in}\;\;\mathrm{dark}}}$$

(10) $$\begin{array}{c} \mathrm{Photochemical}\;\;\mathrm{enhancement}=\frac{C_{\mathrm{CO}2,T_{c},\;\mathrm{with}\;\;\mathrm{light}}-C_{\mathrm{CO}2,T_{c},\;\;\mathrm{in}\;\;\mathrm{dark}}}{C_{\mathrm{CO}2,T_{c},\;\;\mathrm{in}\;\;\mathrm{dark}}} \end{array}$$

where *C*_CO2_ is the CO_2_ conversion, *A* is the gas concentration deduced from the measured peak areas of FIDs, *v* is the gas flow rate, *h* is the reaction time, *V*_M_ is the molar volume of gas, *m* is the catalyst weight, and *S* is the product selectivity.

The photothermal CO_2_ hydrogenation performance of Co_2_C was also evaluated, as listed in Table S2, Supporting Information, in which the catalysts were irradiated with the same 300‐W Xe lamp without external heat.

For the CO hydrogenation reaction, CO_2_ reactant gas was substituted with 1.0 vol% CO/N_2_.

### In Situ DRIFTS Measurements

In situ DRIFTS measurements were performed in a diffuse reflectance cell (Harrick system) equipped with CaF_2_ windows on a Bruker Vertex 70 spectrometer using a mercury–cadmium–telluride (MCT) detector cooled with liquid nitrogen. Before starting, the sample was heat treated in an Ar atmosphere to remove any surface‐interfering molecules. Subsequently, a mixture of 15 vol% CO_2_ (or 2.0 vol% CO), 30 vol% H_2_, and 55 vol% Ar was introduced into the in situ chamber (20 mL min^−1^). The DRIFTS spectra were obtained by subtracting the background from the subsequent spectra. To confirm the reaction pathway, spectra were collected at 300 °C with and without light illumination. The CO_2_/H_2_/Ar mixture was then switched to CO/H_2_/Ar under light illumination to obtain data to support the authors’ hypothesis. Each step was recorded continuously for 20 min until equilibrium was reached.

### Density Functional Theory (DFT) Calculations

DFT calculations were based on a plane‐wave basis set and conducted using the Vienna ab initio simulation package (VASP). The exchange and correlation energies were determined using the ultrasoft pseudopotential and Perdew–Burke–Ernzerhof functions. Electron–on interactions were treated using the projector‐augmented wave (PAW) method. The wave functions were constructed from the expansion of the plane waves with an energy cutoff of 450 eV. Gamma‐centered k‐points of 3 × 3 × 1 were used for geometry optimization. The consistence tolerances for the geometry optimization were set as 1.0 × 10^−5^ eV per atom for the total energy and 0.05 eV Å^−1^ for the force, respectively. To avoid interference between the two surfaces, a vacuum gap of 15 Å was adopted in the periodically repeated slabs. In the free energy calculations, entropic corrections and zero‐point energy (ZPE) were considered.

Typically, the binding energies of the adsorbates on the modeled Co_2_C(111) were calculated as follows:

(11) $$\begin{array}{c} E\left(\mathrm{adsorbates}\right)=E\left(\mathrm{adsorbates}+Co_{2}C\left(111\right)\right)\\ -E\left({\mathrm{Co}}_{2}C\left(111\right)\right)-E\left(\mathrm{adsorbates}\right) \end{array}$$

where *E* (adsorbate + Co_2_C(111)), *E* (Co_2_C(111)), and *E* (adsorbate) are the total energies of the optimized adsorbate + Co_2_C(111), clean Co_2_C(111), and the adsorbate in the gas phase, respectively.

The free energy of species was calculated according to the standard formula:

(12) $$\Delta G=E+\Delta \mathrm{ZPE}+\Delta \delta H_{0}\;-\Delta TS$$

where ZPE is the zero‐point energy, *δH*_0_ is the integrated heat capacity, *T* is the product temperature, and *S* is the entropy.

### Multifunctional Applications

*Photothermal Applications*: The first was photothermal/photodynamic antibacterial therapy. *E. coli*, CMCC(B)44102 and *S. aureus*, CMCC(B)26003 were used as typical bacterial models to perform antibacterial experiments. The spread‐plate count method was used to evaluate the antibacterial performance of the samples. The morphology of the bacteria was observed using an SEM (SU‐70) equipped with an energy dispersive X‐ray spectrometer (EDX). *E. coli* and *S. aureus* were incubated in Luria–Bertani (LB) and Brain Heart Infusion (BHI) media at 37 °C for 18 h. The activated bacterial solutions (1.0 × 10^8^ CFU) were diluted for ten times before use. The aqueous suspensions of Co_2_C were then added to obtain the bacterial solutions (Co_2_C, 200 µg mL^−1^) and the mixture was subsequently irradiated by the simulated solar light (AM 1.5 filter, 0.8 W cm^−2^) for 15 min. Last, the obtained solutions were diluted by 10^4^ times with phosphate buffered saline (PBS), and 30 µL of the mixture was cultured on agar plates at 37 °C for 18 h to quantify the number of colonies. Dehydration was performed to observe bacterial morphology. After centrifugal sedimentation, the treated bacterial solutions were immersed in a 4% paraformaldehyde fixing solution for 30 min and then dehydrated using 25%, 50%, 75%, and 100% ethanol in sequence. Last, the suspension was dispersed on aluminum foil for observation.

Photo‐to‐thermal conversion capacity during antibacterial process was performed by solar light (0.8 W cm^−2^) irradiating the bacterial solutions containing Co_2_C (200 µg mL^−1^), and temperature changes were recorded using an IR thermal imager (FLIR E86).

Reactive oxygen species severely damage microbial cells, deactivating life‐related molecules and eventually inhibiting their activity. The ROS generation capacity of Co_2_C was measured using a transient fluorescence emission spectrum (Edinburgh, FLS1000) with excitation and emission wavelengths of 488 and 525 nm, respectively, using 2’,7’‐dichlorofluorescein diacetate (DCFH‐DA) as an indicator. In brief, 100 µL of the Co_2_C aqueous suspension (1 mg mL^−1^) was mixed with 400 µL of DCFH indicator (100 µmol L^−1^) in a tube, and the mixture was irradiated with solar light (0.8 W cm^−2^) for 15 min for further measurements.

The second application was solar interfacial water evaporation. Before testing, a Co_2_C/PTFE hybrid membrane was prepared by vacuum filtrating 20 mL of Co_2_C aqueous suspension (1 mg mL^−1^) into a PTFE membrane. For the solar interfacial water evaporation performance test, a 100‐mL beaker filled with water was placed on the electronic balance with an accuracy of 0.0001 g, and the Co_2_C/PTFE photothermal membrane was then placed on the top surface of the water. A solar‐light simulator equipped with a standard AM 1.5 solar spectrum was used for the experiment. The light intensity was adjusted to 0.1 W cm^−2^ by the magnitude of the current and calibrated by an optical power meter.

To show the high prospects for photothermal applications of the prepared Co_2_C materials, their photo‐to‐thermal conversion capacity was compared with layered MXenes which are usually regarded as superstars for photothermal purposes. In the test, simulated solar irradiation (0.2 W cm^−2^) illuminated the Co_2_C and referenced MXene powder (20.0 mg), and the temperature changes were recorded using the same IR thermal imager.

*Electrochemical Catalysis Potentials*: This includes hydrogen evolution reaction. Electrochemical tests for the HER were carried out using a conventional three‐electrode cell on a CHI 760E electrochemical workstation at room temperature. The cell for Co_2_C–C_2_H_4_/H_2_ was prepared in a glove box. HER measurements were conducted in 0.5 m of H_2_SO_4_ and 1.0 m of KOH with a graphite‐sheet counter electrode, while calibrated Ag/AgCl and Hg/HgO electrodes were adopted as reference electrodes for the two solutions, respectively. Before the tests, all fresh electrolytes were de‐aerated with argon at room temperature. The working electrode was prepared as follows: an ethanol suspension composed of 800 µL of ethanol, 5 mg of catalyst powder, and 200 µL of 0.5 wt% Nafion solution was obtained by ultrasonic mixing for ≈15 min. Subsequently, 8 µL of the as‐obtained suspension was coated onto a glassy carbon electrode with a diameter of 4 mm. The polarization curves were obtained at room temperature at a scan rate of 5 mV·s^−1^. All potentials were iR‐compensated and converted to the reversible hydrogen electrode (RHE) scale via calibration. All the polarization curves were steady‐state curves after several cycles. CV was conducted in the range from −0.3 to 0.2 V versus RHE at a scan rate of 100 mV·s^−1^ to investigate the durability of the Co_2_C electrocatalyst.

*Energy Storage Potentials*: First, Li–O_2_ battery is discussed. To test the electrochemical Li–O_2_ batteries’ performance, 50 wt% of catalyst, 40 wt% of Super P as the conductive agent, and 10 wt% of polyvinylidene fluoride as the binder were mixed in 10 mL of N‐Methyl pyrrolidone (NMP) under ultrasonication treatment. The resultant slurry was homogeneously dispersed onto carbon paper (TGP‐H‐060, Toray), which was dried at 120 °C under vacuum overnight. Super P cathodes were prepared using a similar procedure, replacing the active material with Super P. The cathodes were assembled with a Li‐foil anode into a 2032‐type coin cell with holes on top in an argon‐filled glovebox (Mbraun). The electrolyte was 1‐m lithium bis(trifluoromethanesulfonyl)imide in triethylene glycol dimethyl ether (LiTFSI/TEGDME). The electrochemical performance of the Li–O_2_ batteries was tested in a dry O_2_‐purged box at room temperature using a multichannel LAND system (CT 2001A). The specific capacities were calculated based on the amount of catalyst prepared in the cathodes. CV was carried out on an electrochemical workstation (RST5002F) at a scan rate of 0.10 mV s^−1^ in the voltage range of 2.0–4.5 V (vs Li^+^/Li). A three‐electrode cell with platinum wire as the counter electrode and a reference electrode in 1‐m LiTFSI/TEGDME was used for the electrochemical active surface area test. The electrochemical impedance spectroscopy (EIS) test was also taken in the same electrochemical workstation with a sine wave of 10 mV and a frequency range of 100 kHz to 0.01 Hz.

Second, Li (Na, K)‐ion batteries are discussed. The electrodes were prepared by mixing 80 wt% Co_2_C, 10 wt% Super P, 10 wt% PVDF, and an appropriate amount of water under homogenous stirring. Next, the prepared slurry was painted on a Cu foil at 80 °C and left overnight under vacuum conditions. Last, the working electrodes were cut into 14‐mm disks with mass loading of ≈0.7–1.1 mg cm^−2^. The assembly process for half‐/full‐cells occurred in an Ar‐filled glove box (both H_2_O and O_2_ lower than 0.1 ppm). For Li‐ion batteries, 1 m LiPF_6_ dissolved in ethylene carbonate/dimethyl carbonate/ethyl methyl carbonate (EC/DMC/EMC, 1:1:1 v/v) was used as the electrolyte. The purchased Li disks and monolayer polypropylene (Celgard 2400) were used as the counter electrodes and separators, respectively. For sodium‐ion batteries (SIBs), 1 m of NaPF_6_ dissolved in diglyme (DME) was used as the electrolyte. Na disks and glass fibers (Whatman GF/D) were used as the counter electrodes and separators, respectively. For potassium‐ion batteries, 1 m of potassium bis(fluorosulfonyl)imide (KFSI) in DME was used as the electrolyte. Freshly made K disks and glass fibers (Whatman GF/D) were used as the counter electrodes and separators, respectively. CV profiles were collected on a CHI760E (Shanghai Chenhua) electrochemical workstation at different scan rates in the region of ≈0.01–3.0 V. The discharge/charge tests were recorded in windows of ≈0.01–3.0 V using a multichannel battery testing system (Land 2001A).

## Conflict of Interest

The authors declare no conflict of interest.

## Supporting information

Supporting Information

## Data Availability

The data that support the findings of this study are available from the corresponding author upon reasonable request.
