# Supplementary material for: Large‐Scale Synthesis of Multifunctional Single‐Phase Co2C Nanomaterials
Source: Adv Sci (Weinh). 2023 Apr 24;10(19):2301073. doi: 10.1002/advs.202301073 (PMC10323641; doi:10.1002/advs.202301073)
Supplement: Supplementary file 1 — Supporting Information [file ADVS-10-2301073-s001.pdf]

ADVANCED SCIENCE
Open Access

Supporting Information

for *Adv. Sci.*, DOI 10.1002/advs.202301073

Large-Scale Synthesis of Multifunctional Single-Phase Co_2C Nanomaterials

*Zhengyi Yang, Tingting Zhao, Shuyan Hao, Rutao Wang, Chunyan Zhu, Yunxiang Tang, Chan Guo, Jiurong Liu, Xiaodong Wen and Fenglong Wang**

Supporting information

Large-scale Synthesis of Multifunctional Single-Phase Co_2C Nanomaterials

Zhengyi Yang[a], Tingting Zhao[a], Shuyan Hao[a], Rutao Wang[a], Chunyan Zhu[a], Yunxiang Tang[a], Chan Guo[a], Jiurong Liu[a], Xiaodong Wen[b,c,d], Fenglong Wang[a,e,]*

[a] Key Laboratory for Liquid-Solid Structural Evolution and Processing of Materials Ministry of Education, Shandong University, Jinan 250061, P. R. China

[b] State Key Laboratory of Coal Conversion, Institute of Coal Chemistry, Chinese Academy of Sciences, Taiyuan, 030001 P. R. China

[c] National Energy Center for Coal to Liquids, Synfuels China Co. Ltd. Huairou District, Beijing, 101400 P. R. China

[d] Beijing Advanced Innovation Center for Materials Genome Engineering, Beijing Information S & T University, Beijing, 101400 P. R. China

[e] Shenzhen Research Institute of Shandong University, Shenzhen, Guangdong, 518057, China

***Corresponding Authors' Emails:**

fenglong.wang@sdu.edu.cn (F.L.W)

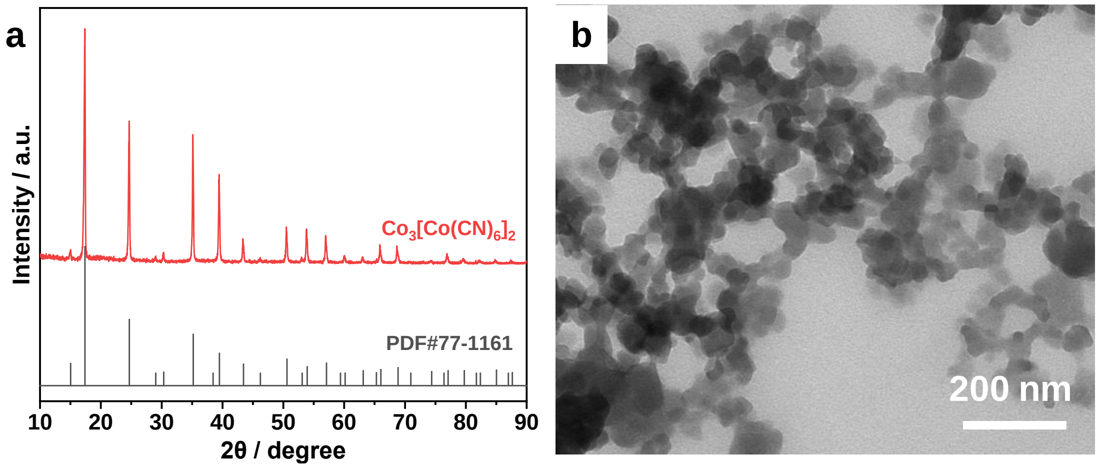

Figure S1. a) XRD pattern, and b) TEM image of prepared Co-PBA precursor.

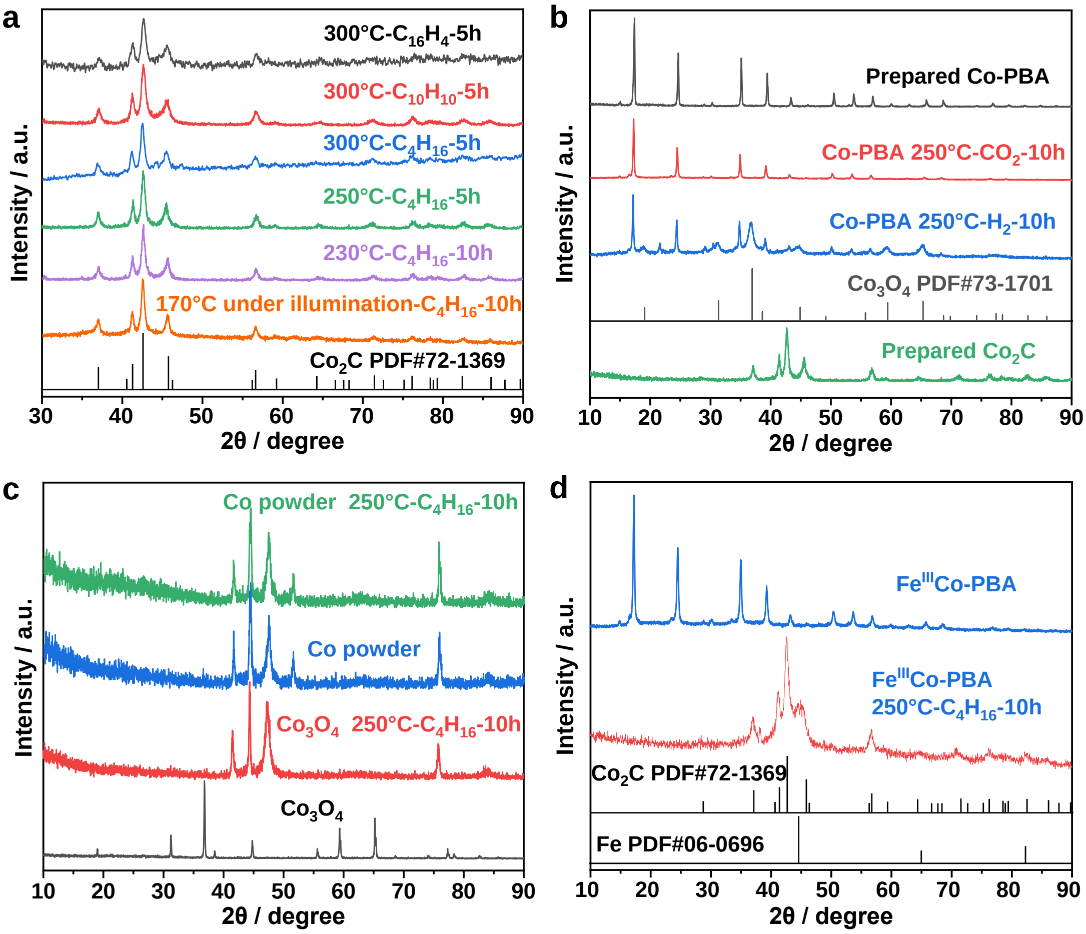

Figure S2. a) XRD patterns of the Co_2C materials prepared under varied CO_2/H_2 ratios, temperatures with/without light irradiation. For example, the 250°C-C_4H_{16}-5h described the sample prepared at 250 °C with CO_2/H_2 flow rates of 4/16 sccm for 5 hours; b) XRD patterns of the Co-PBA precursor calcined under sole H_2 and CO_2 stream; c) XRD patterns of the purchased Co and Co_3O_4 powder before/after carburizing process; d) XRD patterns of Fe^{3+} doped Co-PBA under CO_2/H_2 gas mixture atmosphere.

Notes:

Figure S2a indicated that the varied CO_2/H_2 ratios ranging from 4:1~1:4 did not

affect the crystalline structure of prepared Co_2C. The minimum carburization temperature was about 230 °C in dark and 170 °C with light irradiation. **Figure S2b** indicated that both the CO_2 and H_2 gas were indispensable for Co_2C formation. **Figure S2c** indicated that the traditional metal/metal oxides precursors could not be converted to Co_2C, in the same carbonization condition, reflecting the advantage of Co-PBA precursor for Co_2C production. As a special class of coordinated frameworks, the Co atoms in PBA could be replaced partially or entirely by other common transition metals and noble metals,[1] and **Figure S2d** hinted the considerable potential in the preparation of various metal carbides derived from PBA with tunable compositions (herein, Fe-doped Co_2C materials), which is still under investigation in our group.

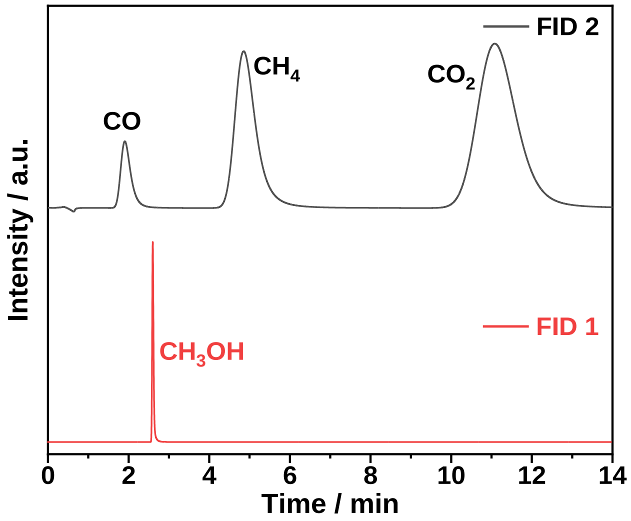

Figure S3. GC spectra of the gaseous products during Co_2C nanomaterials formation in CO_2/H_2 gas mixture atmosphere.

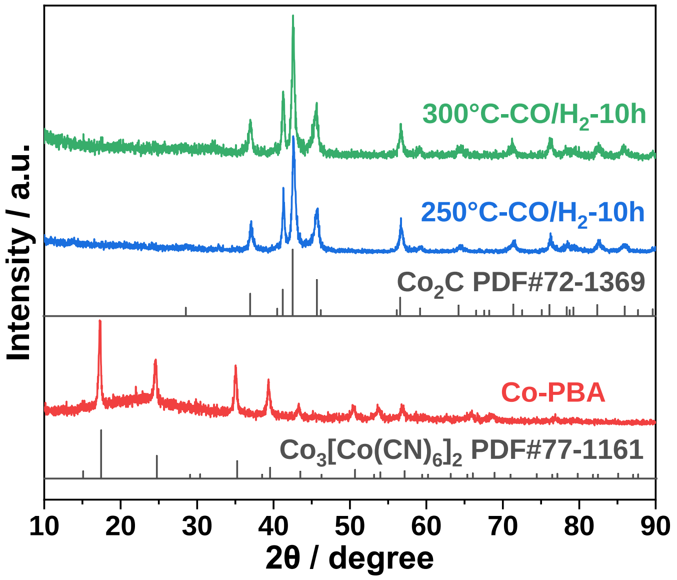

Figure S4. XRD patterns of the Co_2C materials prepared using CO/H_2 at different temperatures. For example, the 250°C-CO/H_2-10h described the sample prepared at 250 °C with CO/H_2 flow rates of 10/10 sccm for 10 hours.

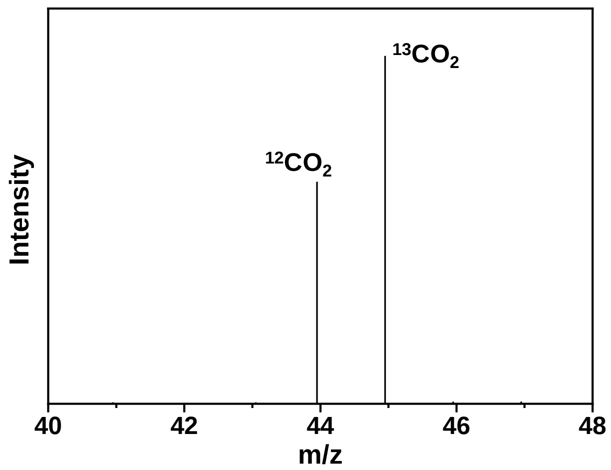

Figure S5. The MS spectrum of the collected CO_2 gas samples from Co_2C oxidation at 400 °C after switching $^{13}CO_2/H_2$ flow to O_2 for 10 min.

Notes:

The Carbon-13 isotope tracing experiments were conducted in the miniature photo-assisted thermal catalytic micro reactor. To figure out the source of the carbon element in Co_2C, the Co-PBA was directly added into the photo-assisted micro reactor and heated to 300 °C under light irradiation and maintained for 10 h in an Ar atmosphere to remove the surface interfering molecule. Subsequently, a mixture of $^{13}CO_2/H_2$ (4/16 sccm) was introduced into the reactor and the compositions of gas samples from the outlet of reactor were analyzed by an online GC. After GC spectra stabilized for 5 h (considered that the Co-PBA has entirely transformed into Co_2C, confirmed in **Figure S2a**), the gas samples were collected repeatedly for component analysis. Then, the $^{13}CO_2/H_2$ flow was switched to O_2 and the reactor temperature was increased to 400 °C to completely oxidize the *in-situ* formed Co_2C into cobalt oxide and

carbon dioxide gas. The CO_2 samples were collected as well. **Figure S5** indicated that the carbon element in the *in-situ* formed Co_2C was dominantly ^{13}C labelled, confirming the carbon element in Co_2C originated from both $^{13}CO_2$ gas and Co-PBA (^{12}C) precursor.

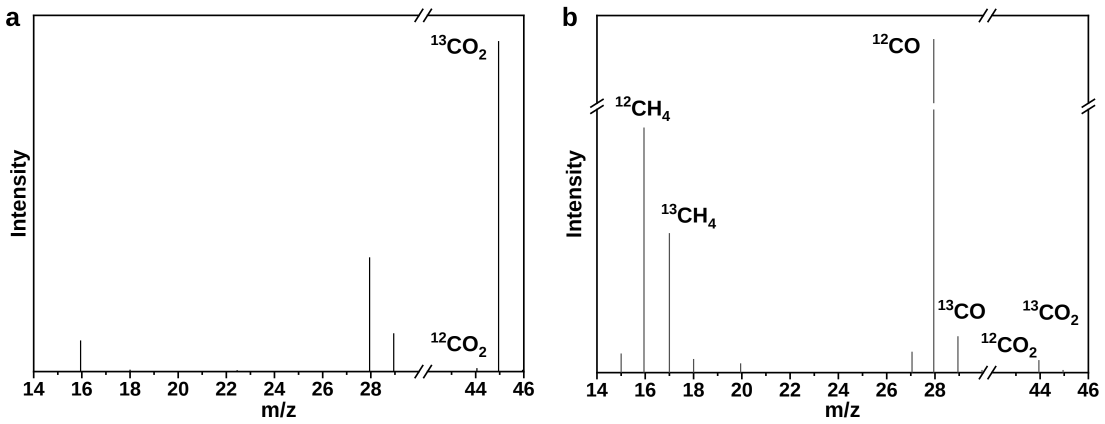

Figure S6. The MS spectra of a) the purchased 99.0 atom% $^{13}CO_2$, and b) the collected samples from the reactor outlet at 300 °C under light irradiation with a $^{13}CO_2/H_2$ stream after 5 h.

Notes:

In terms of carbon element, as shown in **Figure S6a**, the reactant was $^{13}CO_2$. However, after hydrogenation process, the ^{12}C labelled products were more dominant compared with the ^{13}C labelled products (**Figure S6b**). Significantly, the CO_2 redundant from the reactor outlet was primarily ^{12}C labelled (m/z = 44) rather ^{13}C labelled (m/z = 45), meaning that a dynamic chemical exchange process between $Co_2{}^{12}C$ and $Co_2{}^{13}C$ existed during the reaction. As the carburization proceeds, the ^{12}C element in Co_2C was gradually substituted by ^{13}C element, and the results were also in line with the observations in **Figure S5**.

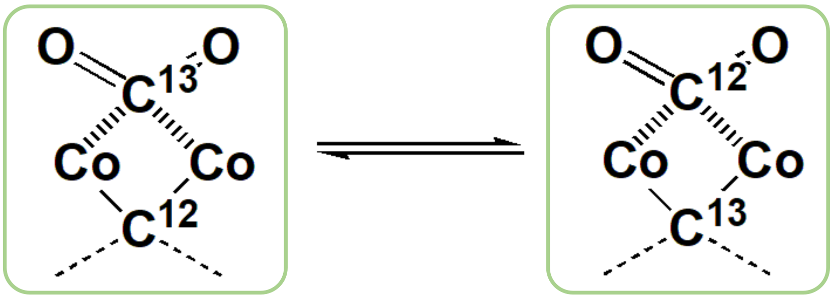

Figure S7. The proposed dynamic chemical equilibrium scheme of adsorbed CO_2 on Co_2C during the CO_2 hydrogenation.

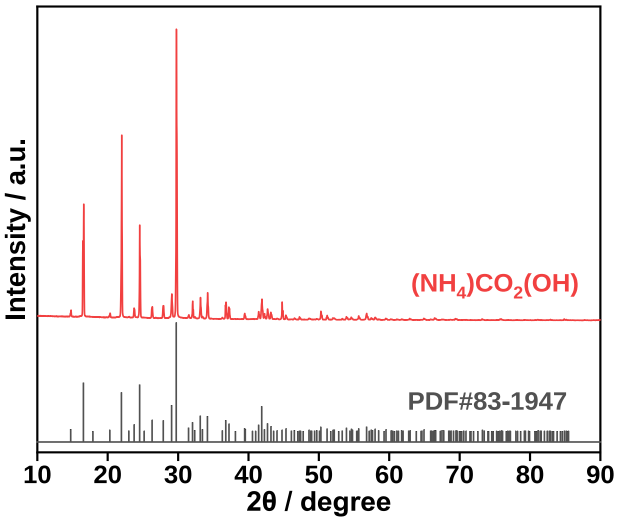

Figure S8. XRD pattern of $(NH_4)CO_2(OH)$ product collected after the Co_2C was prepared at the outlet of the reactor.

Notes:

We scratched the white powders at the outlet of the reactor after Co_2C preparation and analyzed them using XRD to confirm the compositions. It is found that $(NH_4)CO_2(OH)$ was produced accompanied with the formation of Co_2C.

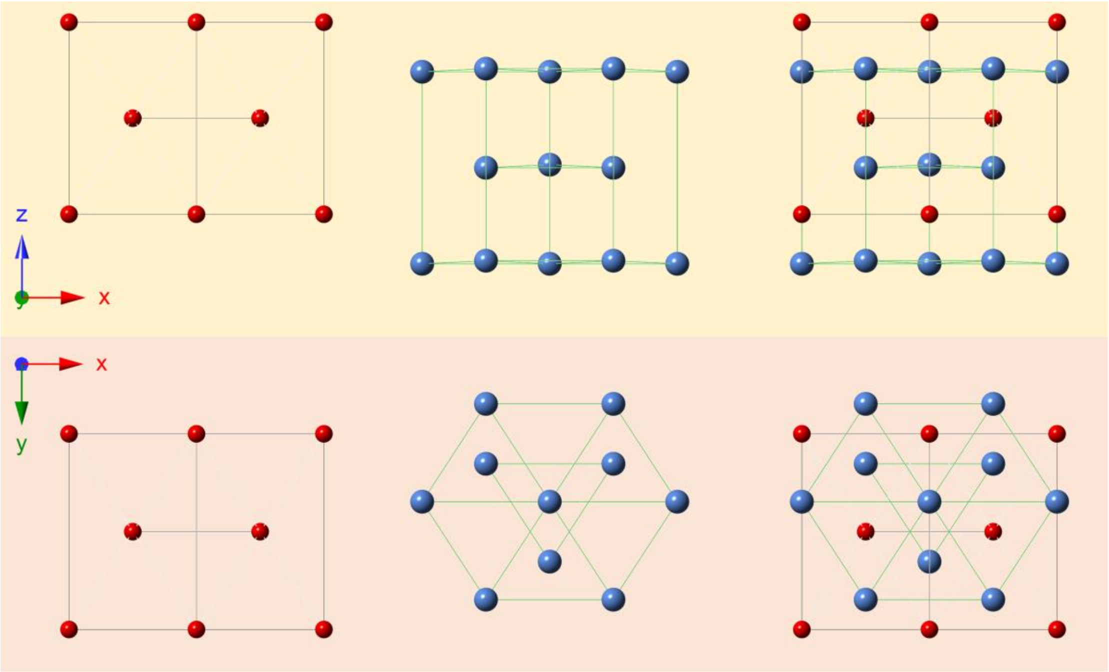

Figure S9. Schematic crystal structure of Co_2C.

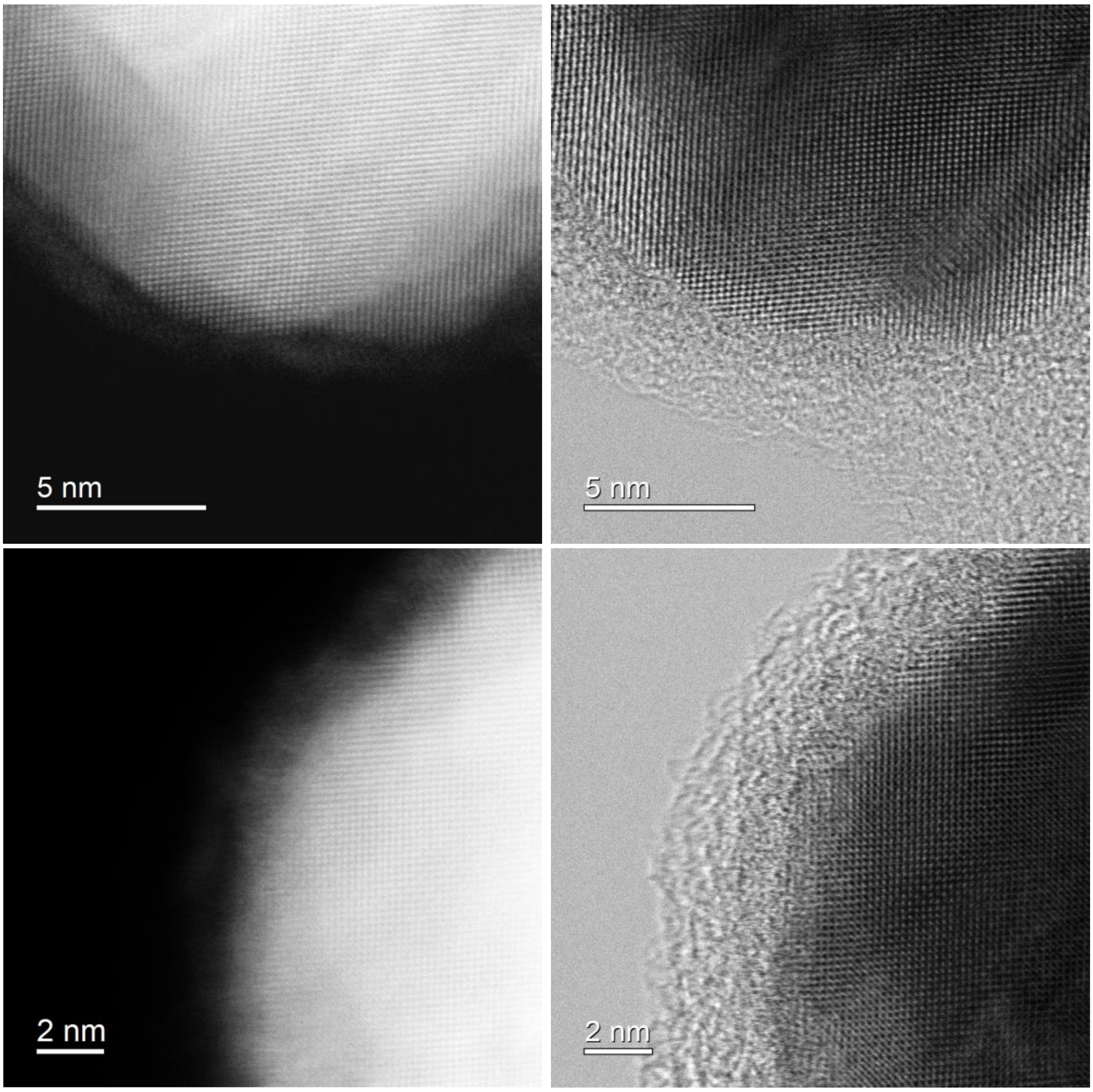

Figure S10. HAADF-STEM images of Co_2C.

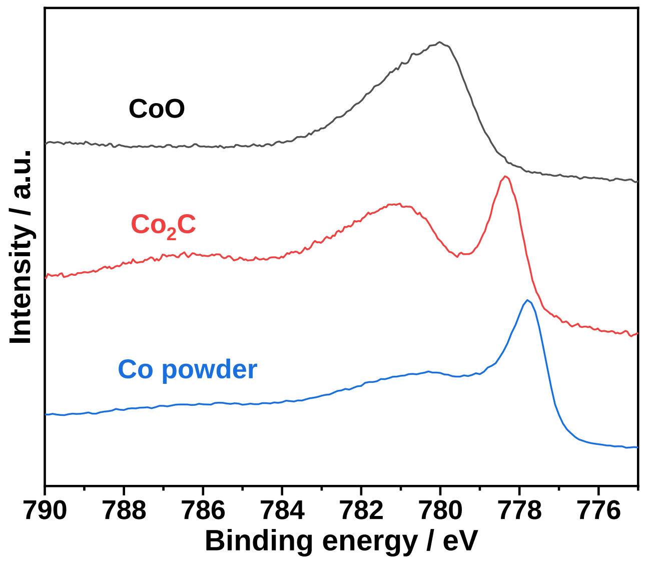

Figure S11. Co $2p_{3/2}$ XPS spectra of purchased CoO, Co powder, and prepared Co_2C materials.

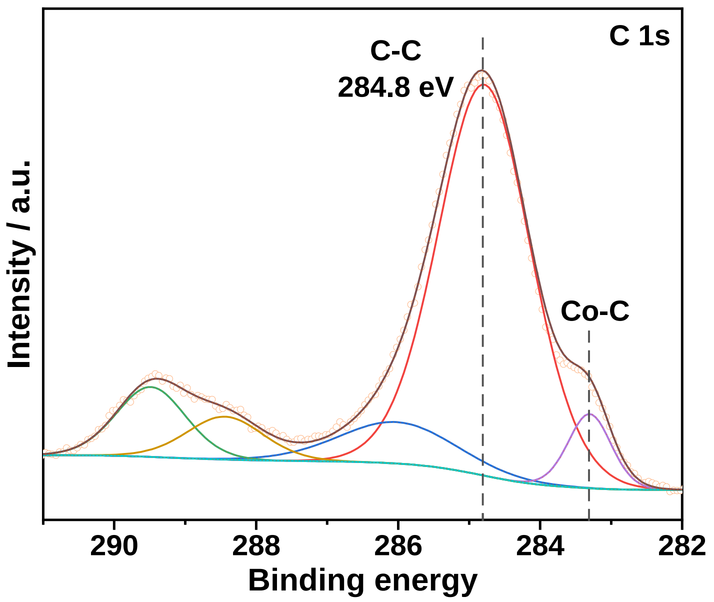

Figure S12. C 1s XPS spectrum of Co_2C.

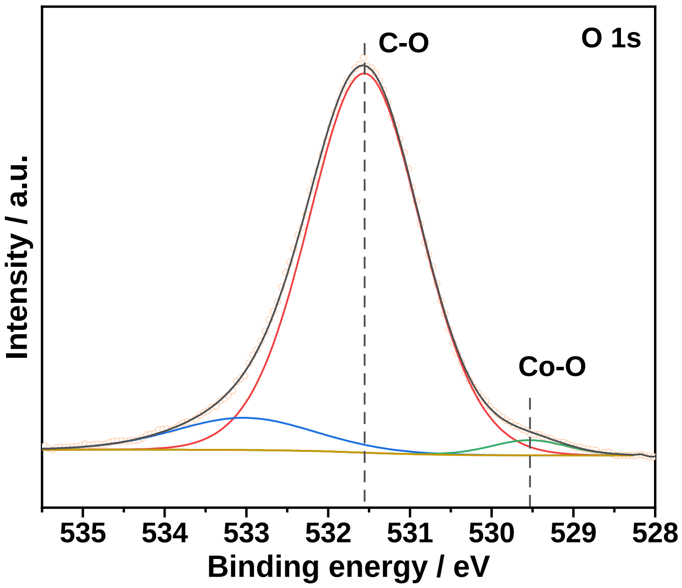

Figure S13. O 1s XPS spectrum of Co_2C.

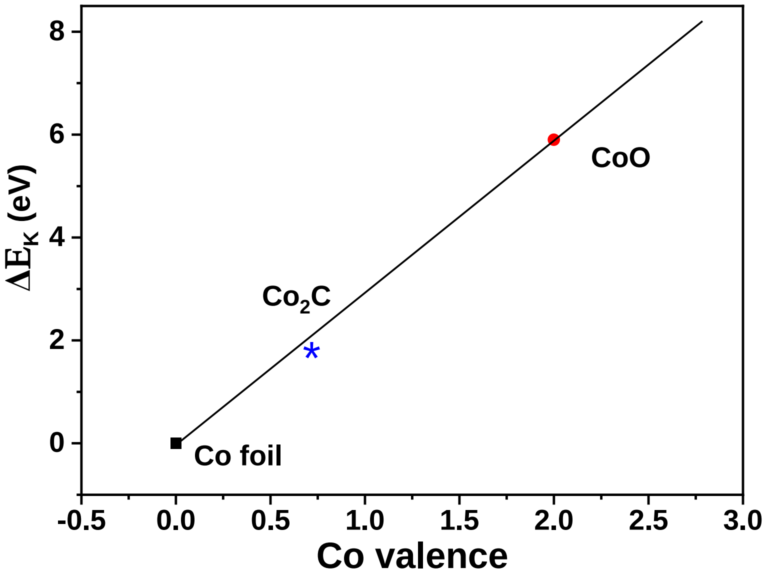

Figure S14. Estimated Co valence state as a function of energy shift.

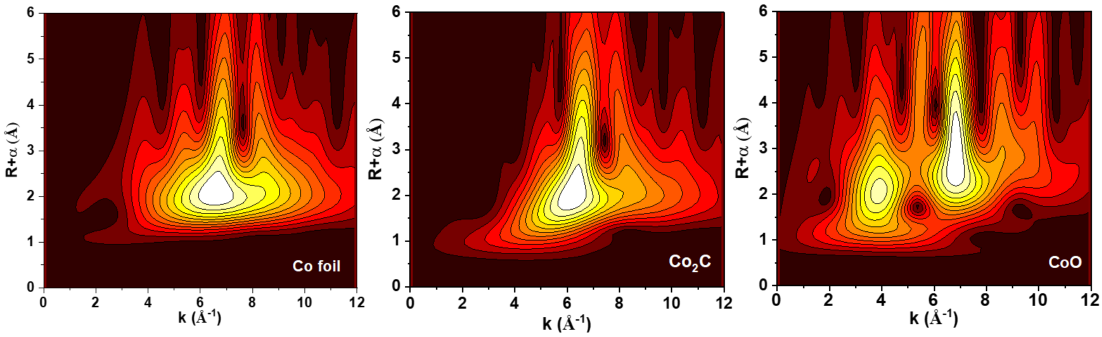

Figure S15. WT-EXAFS analysis of Co foil, Co_2C, and CoO.

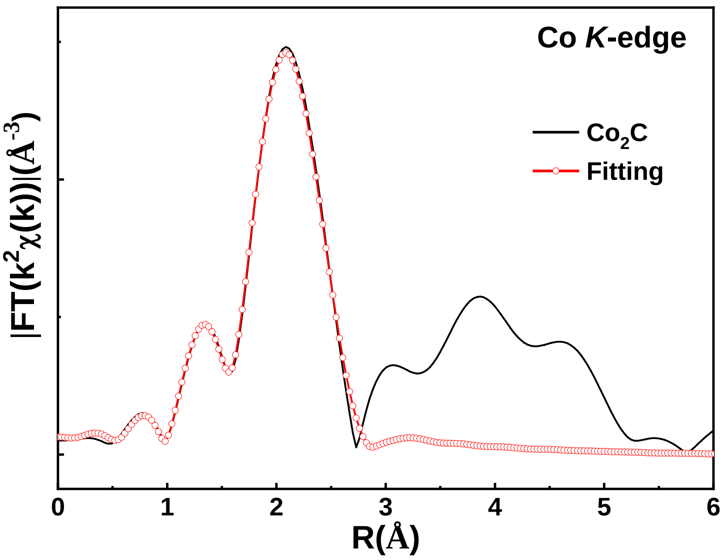

Figure S16. EXAFS fitting result for Co K-edge of Co_2C.

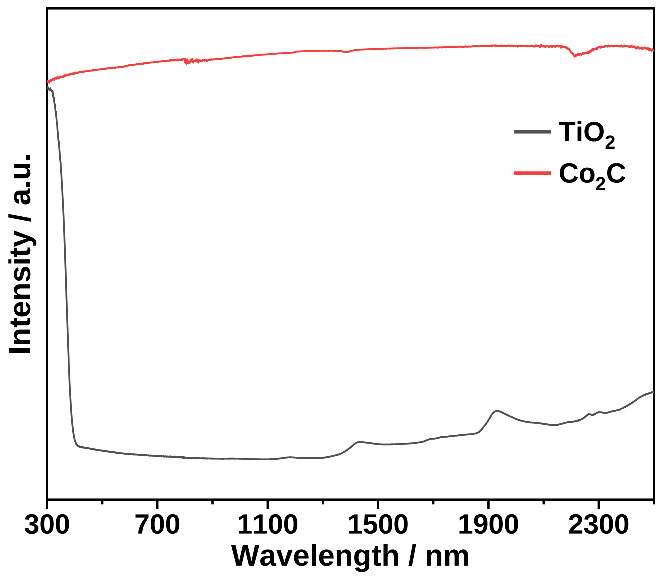

Figure S17. Diffuse reflectance spectra of prepared Co_2C and referenced anatase TiO_2 nanoparticles.

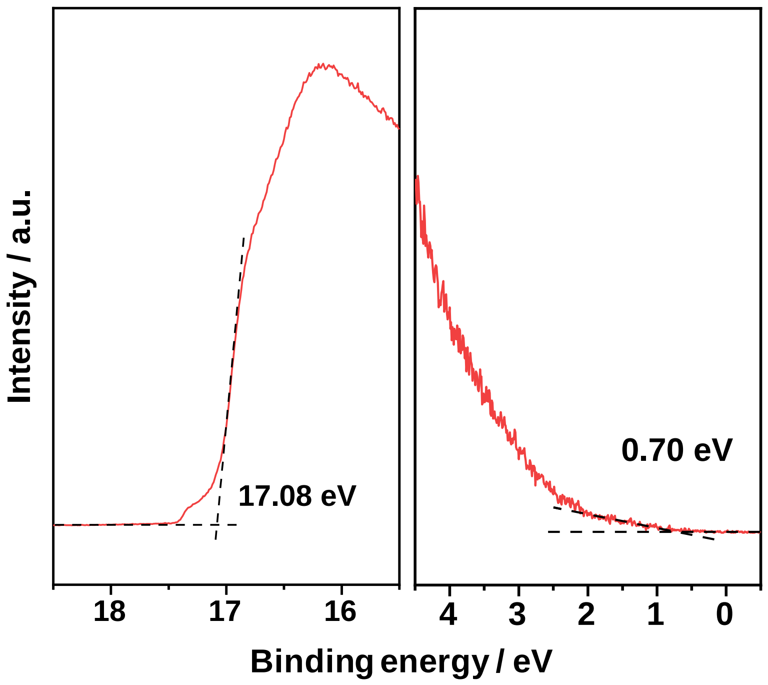

Figure S18. UPS spectra of Co_2C measured by He I (hv = 21.22 eV), and the work function (Φ) = hv − I E_{cutoff} − E_F I.

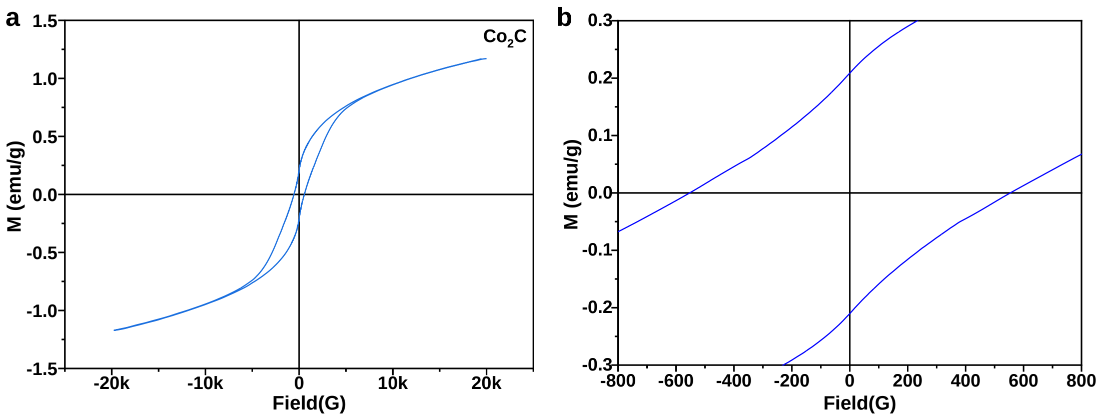

Figure S19. a-b) Magnetic hysteresis loops of the Co_2C.

Notes:

As depicted in **Figure S19**, the prepared Co_2C powder exhibited superparamagnetic behavior without saturation magnetization, which agrees with previous reports,[2] further demonstrating the high purity of Co_2C materials in this work and excluding the existence of metallic Co and Co_3C species in the samples.[3]

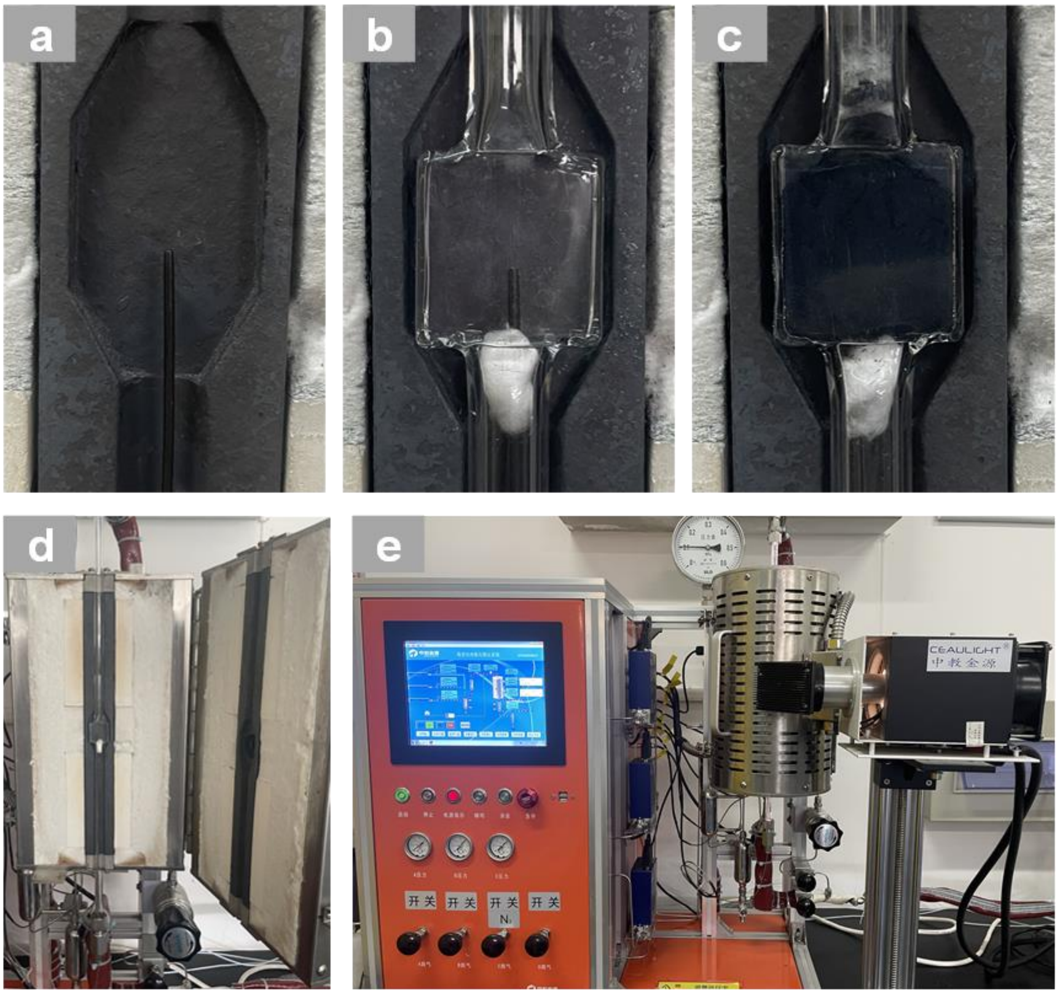

Figure S20. Digital images of miniature photo-assisted thermal catalytic micro reaction system: a) the thermocouple location; b) the quartz reactor; c) the reactor filled with catalyst; d) the external heating furnace; e) its front view.

Notes:

In this work, for the photo-assisted thermal catalytic process, heat was provided by the furnace, and the corresponding set reactor temperature was denoted as Te. The actual catalyst surface temperature, denoted as Tc, was measured by the thermocouple, which inserted into the middle of catalyst layer (ca. 0.25 mm

to the catalysts surface). The inner dimensions of the quartz flow reactor were 24 mm × 24 mm × 2 mm, and the diameter of the thermocouple was 1.5 mm. In dark, Tc = Te. Under light illumination, owing to the photothermal effect of the catalyst, Tc was always higher than Te.

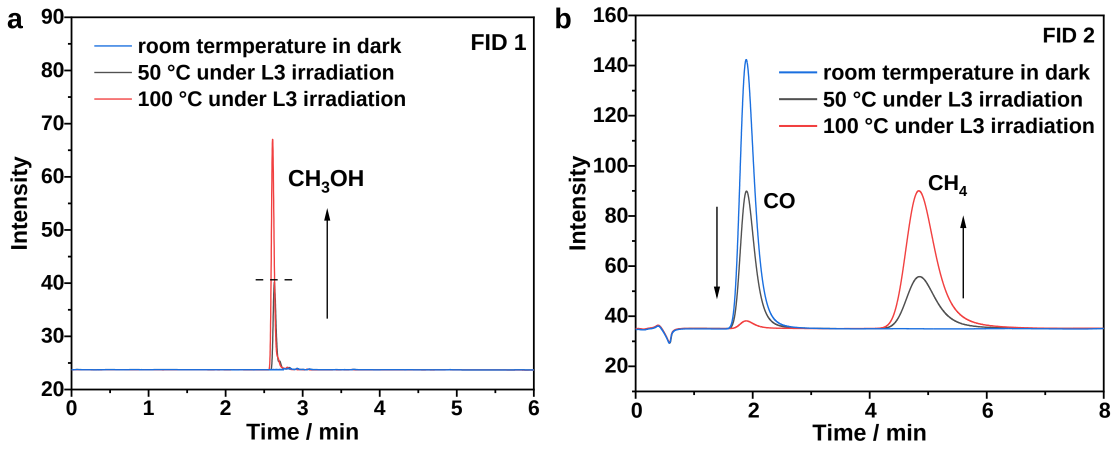

Figure S21. GC spectrums of products during photo-assisted thermal CO hydrogenation over Co_2C: a) FID 1 and b) FID 2 under different temperature with/without light illumination.

Notes:

The photo-assisted thermal CO hydrogenation performance over Co_2C is shown in **Figure S21**. Under light irradiation, the Co_2C catalyst efficiently catalyzed CO hydrogenation to CH_4 with high production selectivity, confirming the feasibility of the hydrogenation of adsorbed CO (*CO) to CH_4 under illumination.

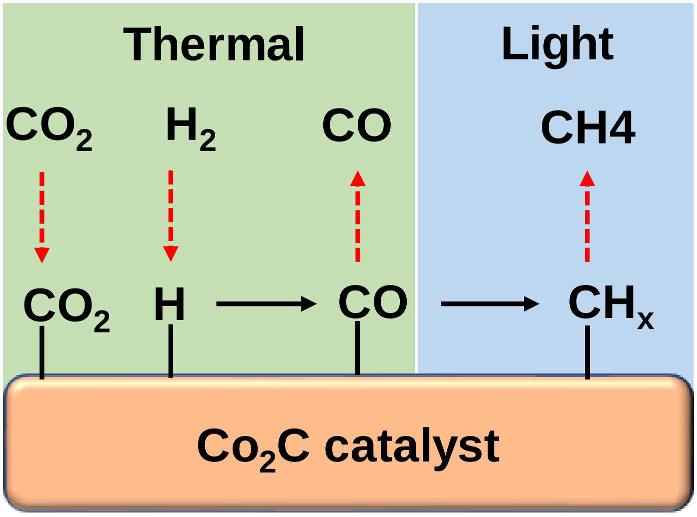

Figure S22. The proposed reaction pathway of Co_2C catalyst for RWGS and CH_4 formation under photo-assisted thermal catalytic process.

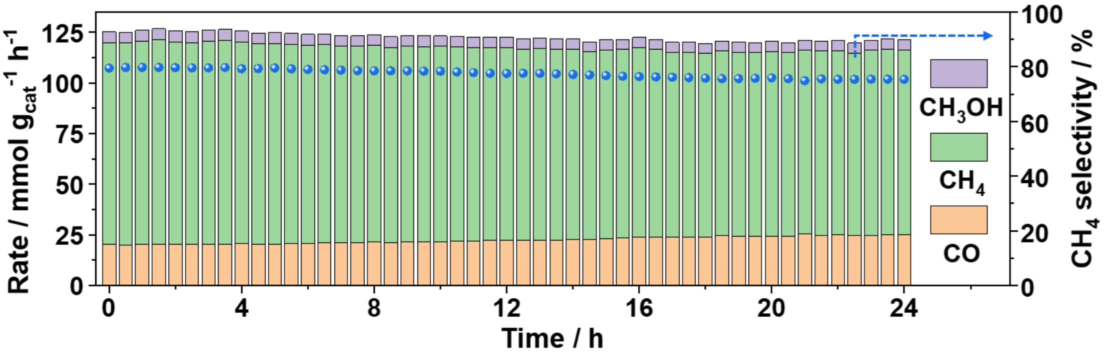

Figure S23. Twenty-five-hour continuous stability test of Co_2C for photo-assisted thermal CO_2 hydrogenation at Te = 300 °C with L3 light irradiation.

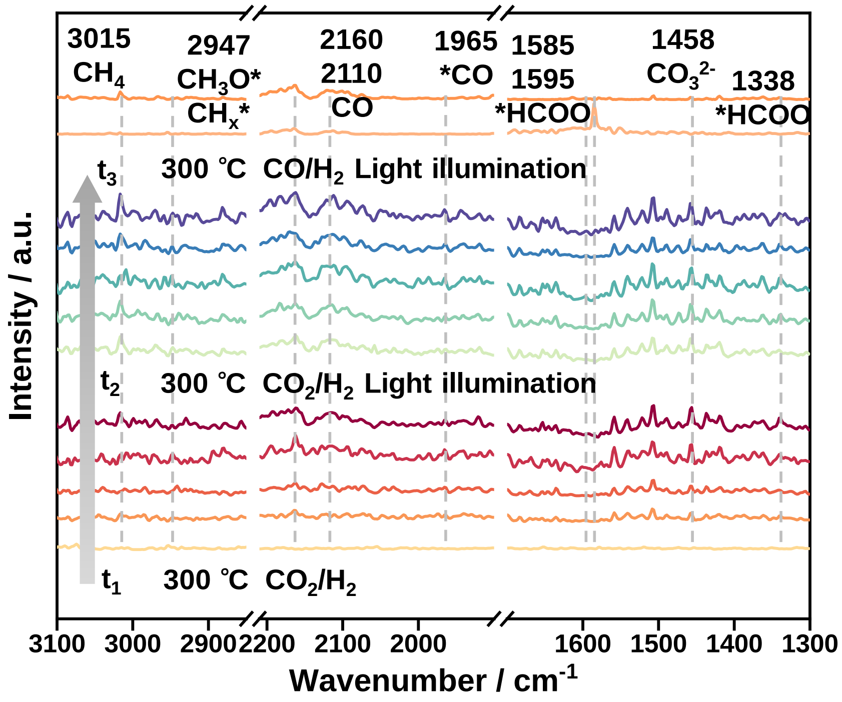

Figure S24. *In-situ* DRIFTS spectra obtained on Co_2C under successively varied conditions.

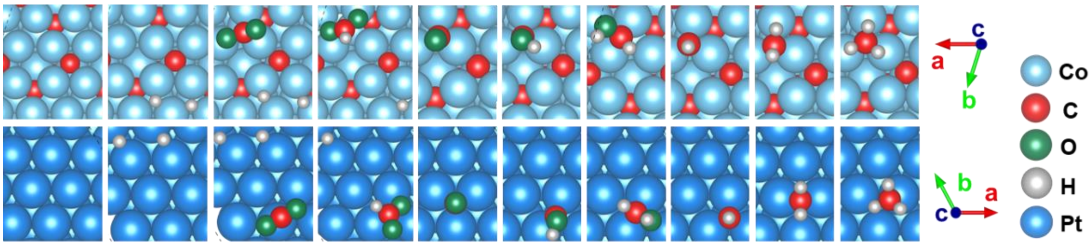

Figure S25. Molecular-level mechanism from DFT calculations for CO_2 hydrogenation into methane.

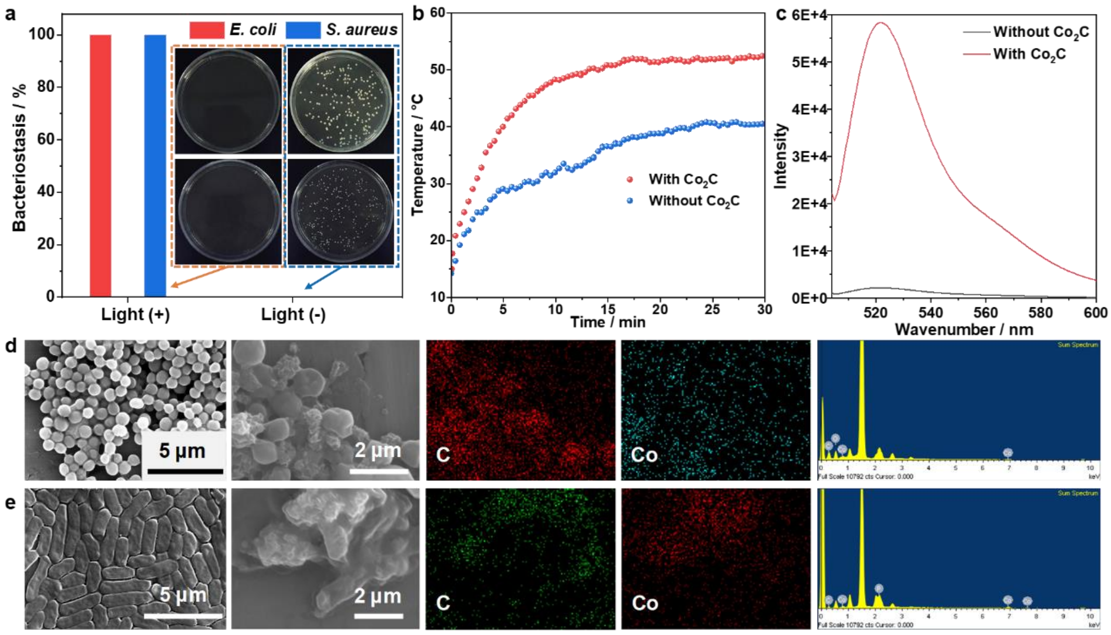

Figure S26. Photothermal/photodynamic antibacterial performances of Co_2C materials. a) Antibacterial efficiency of the Co_2C against *E. coli* and *S. aureus*; b) Temperature elevation curve of bacterial solutions with/without Co_2C as a function irradiation time; c) Fluorescence emission spectra of Co_2C with reference H_2O; SEM images and corresponding EDX results of d) *E. coli* and e) *S. aureus* before/after light irradiation for 15 min treated with 200 μg/mL of Co_2C.

Notes:

As shown in **Figure S26a**, under 8 suns light irradiation, the Co_2C materials achieved nearly 100% inactivation against *S. aureus* and *E. coli* within 15 min. Photo-to-thermal conversion tests in **Figure S26b** indicated that bacterial solutions containing Co_2C under light illumination could rapidly reach approximately 52 °C. In addition to the excellent photo-to-thermal conversion

performance, as shown in **Figure S26c**, the high ROS generation was another key factor for the enhanced antibacterial efficiency. The morphologies and membrane integrity of the bacteria treated before and after light irradiation are shown in **Figure S26d–e**. Briefly, after culturing with Co_2C under light irradiation, the cells exhibited considerable damage and shriveled and cracked, indicating a high bactericidal efficacy.

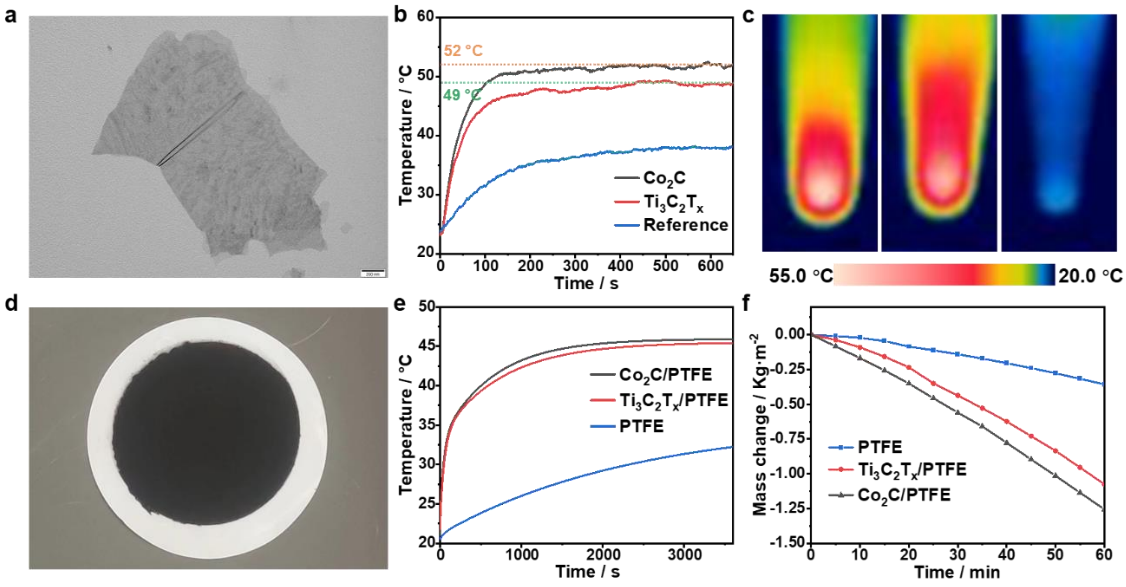

Figure S27. Solar-driven interfacial water evaporation performances. a) TEM image of few-layered MXenes; b) Photo-to-thermal conversion comparison of prepared Co_2C materials and few-layered MXenes, and c) their corresponding IR thermal images. The weight of samples was 20.0 mg; d) Digital image of Co_2C/PTFE membrane; e) The temperature curses and f) water evaporation performance of samples as a function of irradiated time. Light source: one sun light intensity.

Notes:

As shown in **Figure S27b–c**, under two suns of light irradiation, the surface temperatures of the Co_2C powder and MXene reached 52 and 49 °C, respectively. Surprisingly, compared to few-layered MXenes (which are considered as “superstars” for photothermal applications), Co_2C exhibited a higher photo-to-thermal conversion ability. Owing to its robust capacity for generating heat using light irradiation, the Co_2C/PTFE sample achieved a water

evaporation rate of 1.26 kg m^{-2} h^{-1} (**Figure S27f**) under one solar light, which exceeded that of the reference $Ti_3C_2T_x$/PTFE sample (1.07 kg m^{-2} h^{-1}).

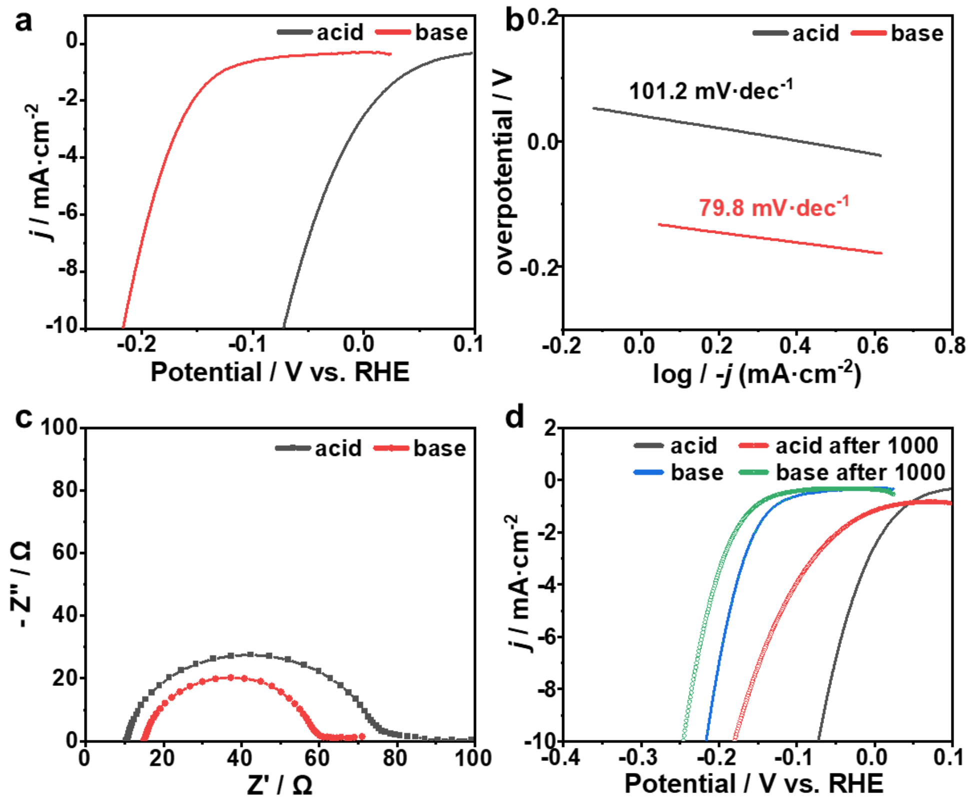

Figure S28. Electrochemical hydrogen evolution reaction performance. a) Polarization curves of Co_2C electrocatalyst with 1 M of KOH and 0.5 M of H_2SO_4 electrolytes; b) corresponding Tafel plots and c) EIS plots for Co_2C electrocatalysts; d) Polarization curves for the Co_2C catalyst after 1000 cycles.

Notes:

As the electrocatalyst for hydrogen evolution reaction, Co_2C materials exhibited high electrocatalytic activity. As shown in **Figure S28a**, under a cathodic current density of 10 mA·cm^{-2}, the overpotential reached 75 mV with a 0.5-M H_2SO_4 electrolyte and 216 mV with a 1.0-M KOH electrolyte. The fitted Tafel slopes of Co_2C reached 79.8 and 101.2 mV·dec^{-1} with base and acid electrolytes (**Figure S28b**). EIS tests revealed a low charge transfer resistance

during the electrochemical reaction (**Figure S28c**). After 1000 cyclic voltammetry cycles, the Co_2C catalyst in basic solution exhibited good activity and durability (**Figure S28d**).

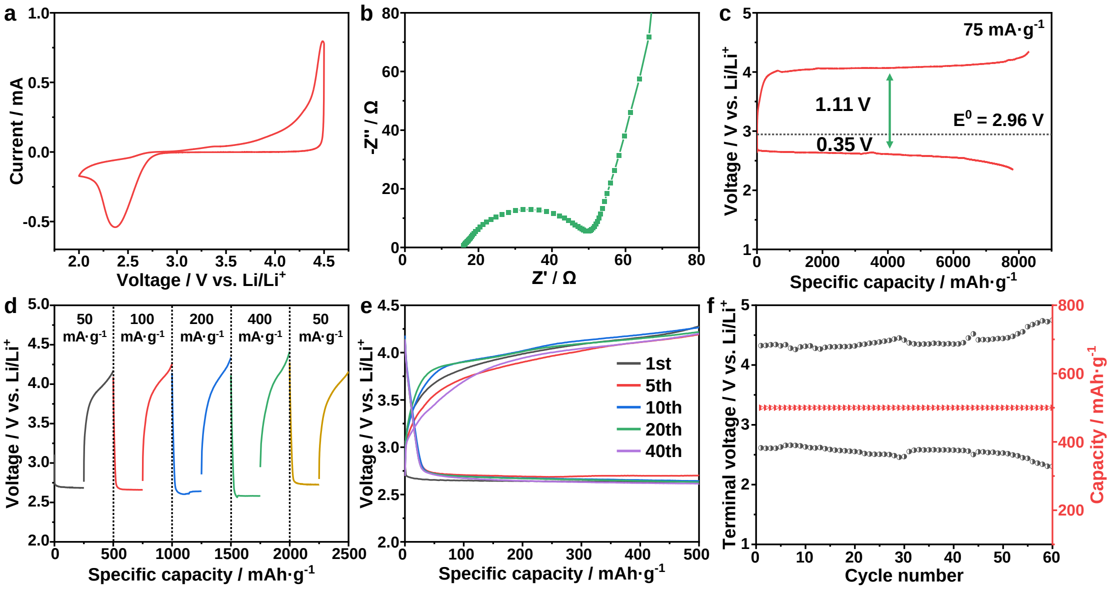

Figure S29. Electrochemical performances for Li-O_2 battery with Co_2C cathode materials. a) CV curve of Co_2C cathodes at 0.10 mV s^{-1} within a voltage window of 2.0–4.5 V; b) EIS plots; c) initial discharge/charge profiles of Co_2C cathodes at 75 mA g^{-1} from 2.35 to 4.35 V; d) rate capability of Co_2C cathodes; e) selected discharge/charge profiles of the Co_2C cathodes with fixed specific capacities of 500 mAh g^{-1} from cycling performance; and f) cycling performance of Co_2C cathodes at 100 mA g^{-1}.

Notes:

As shown above, pristine Co_2C exhibited a large overpotential as a Li-O_2 battery cathode material. The CV curves in **Figure S29a** with a large current density and integral area indicate good electrocatalytic activity of Co_2C materials, and the EIS plots in **Figure S29b** with a small semicircle diameter indicate good electronic conductivity. As shown in **Figure S29c**, the initial full

discharge/charge profiles confirmed the good output capacity, and at a current density of 75 mA g^{-1}, Co_2C delivered a specific capacity of 7814 mAh g^{-1}, which is close to that of the reported MXene materials.[4] The rate performances of Co_2C at a fixed specific capacities of 500 mAh g^{-1} in **Figure S29d** suggested that with the rise in current density from 50 to 400 mA g^{-1}, the charge potential plateau of Co_2C increases from 4.17 to 4.42 V and recovers back to 4.16 V when the current is switched back to 50 mA g^{-1}, reflecting the favorable reversibility. The Co_2C cathode also delivered excellent cycling performance, with stable terminal voltages for 60 cycles, suggesting good electrocatalytic stability (**Figure S29e–f**).

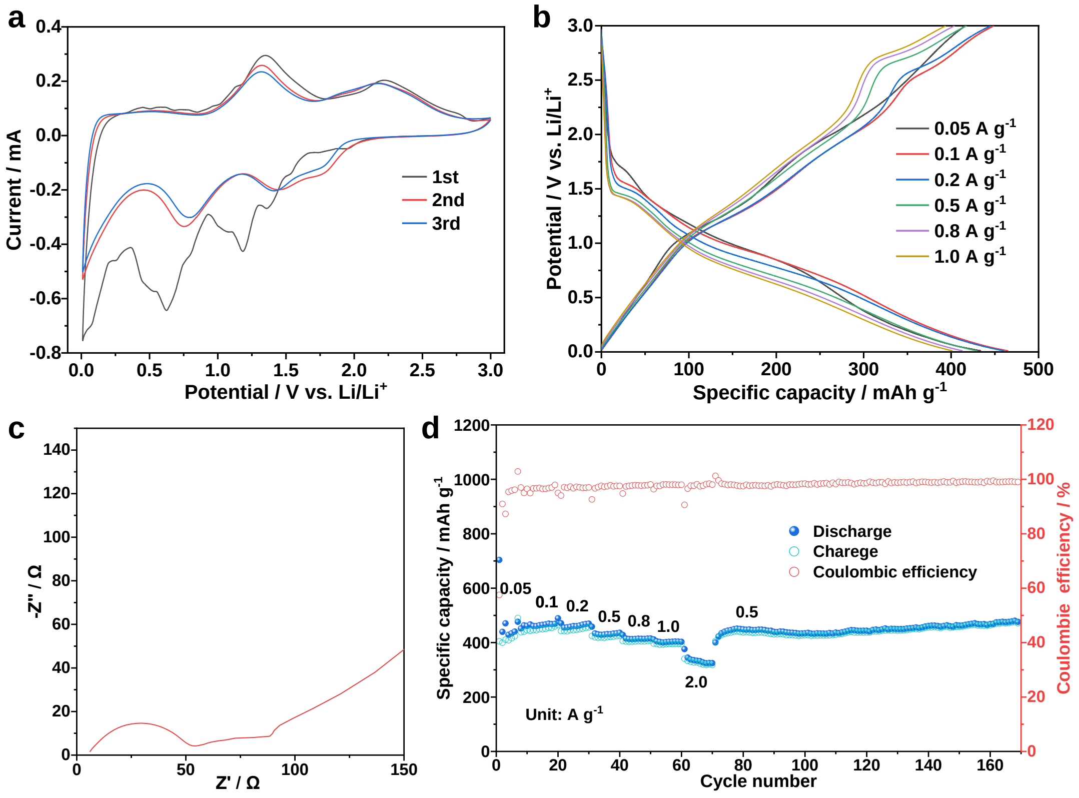

Figure S30. Electrochemical performances for Li-ion battery with Co_2C materials. a) CV curves for the first three cycles of Co_2C electrode at 0.2 mV s^{-1}; b) selected charge-discharge profiles of Co_2C electrode at varied current density; c) electrochemical impedance spectra of Co_2C electrode; and d) rate capacities and cycling performance of Co_2C electrode at varied current density.

Notes:

As shown in **Figure S30a**, the profile of the initial cycle in CV with peaks was different from those of the subsequent ones because of the formation of solid electrolyte interface (SEI) films with the preliminary decomposition of the electrolyte and other irreversible reactions.[5] During the subsequent anodic scan, the overlapped cathodic and anodic peaks indicated abundant redox

reactions with excellent reversibility and stability of the electrode. The selected charge-discharge profiles at various current densities indicated good cyclic stability of the Co_2C electrode (**Figure S30b**). The EIS plot with a small semicircle diameter indicates good electronic conductivity of the Co_2C materials (**Figure S30c**). Co_2C achieved a superior rate performance of 0.05 to 2 A g^{-1}. As depicted in **Figure S30d**, the specific capacities reached 440.4, 465.3, 460.1, 430.9, 414.4 and 395.0 mAh g^{-1}, when the current densities increased from 0.05, 0.1, 0.2, 0.5, 0.8 to 1.0 A g^{-1}, respectively. In particular, the specific capacity also returns to a similar value to the initial one at a current density of 0.5 A g^{-1}, suggesting good reversibility. After 100 cycles, the reversible capacity was retained at 476.1 mAh g^{-1} with a coulombic efficiency of 99.0%, indicating a good cycling performance.

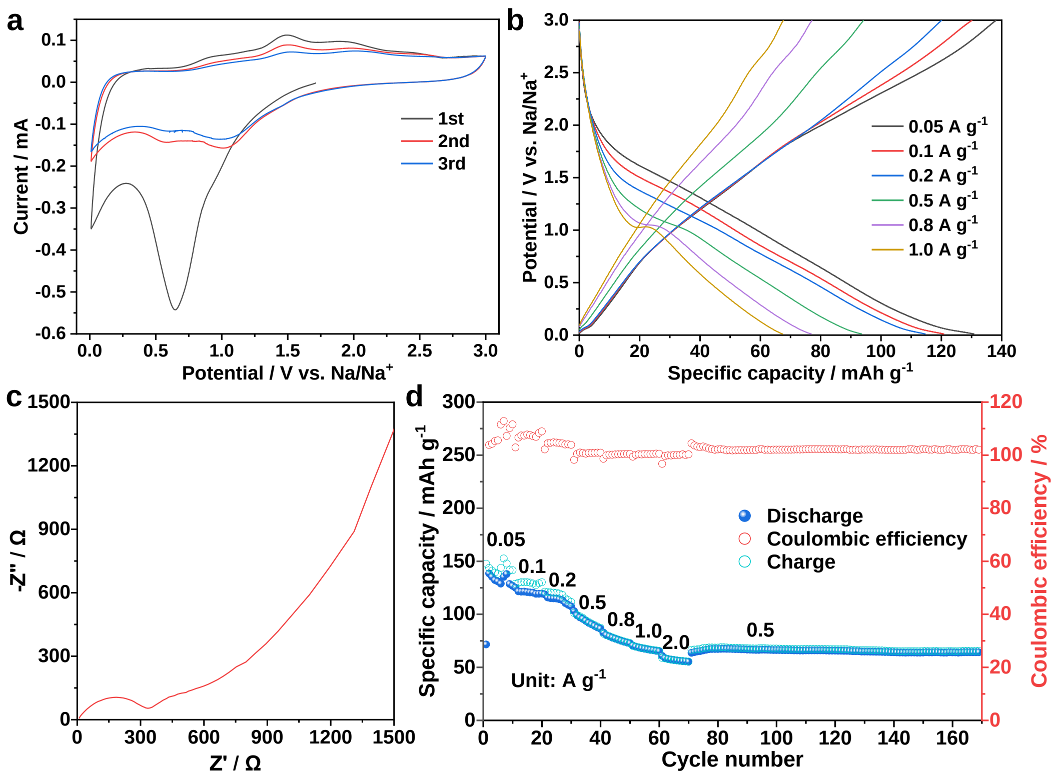

Figure S31. Electrochemical performances for Na-ion battery with Co_2C materials. a) CV curves for the first three cycles of Co_2C electrode at 0.2 mV s^{-1}; b) selected charge-discharge profiles of Co_2C electrode at varied current density; c) electrochemical impedance spectra of the Co_2C electrode; and d) rate capacities and cycling performance of Co_2C electrode at varied current density.

Notes:

Co_2C achieved a good rate performance at different current densities from 0.05 to 2 A g^{-1}. The specific capacities reached 132.9, 122.5, 114.5, 93.9, 77.5 and 69.0 mAh g^{-1}, when the current densities increased from 0.05, 0.1, 0.2, 0.5, 0.8 to 1.0 A g^{-1}, respectively, with good reversibility, cycling performance, and high coulombic efficiency.

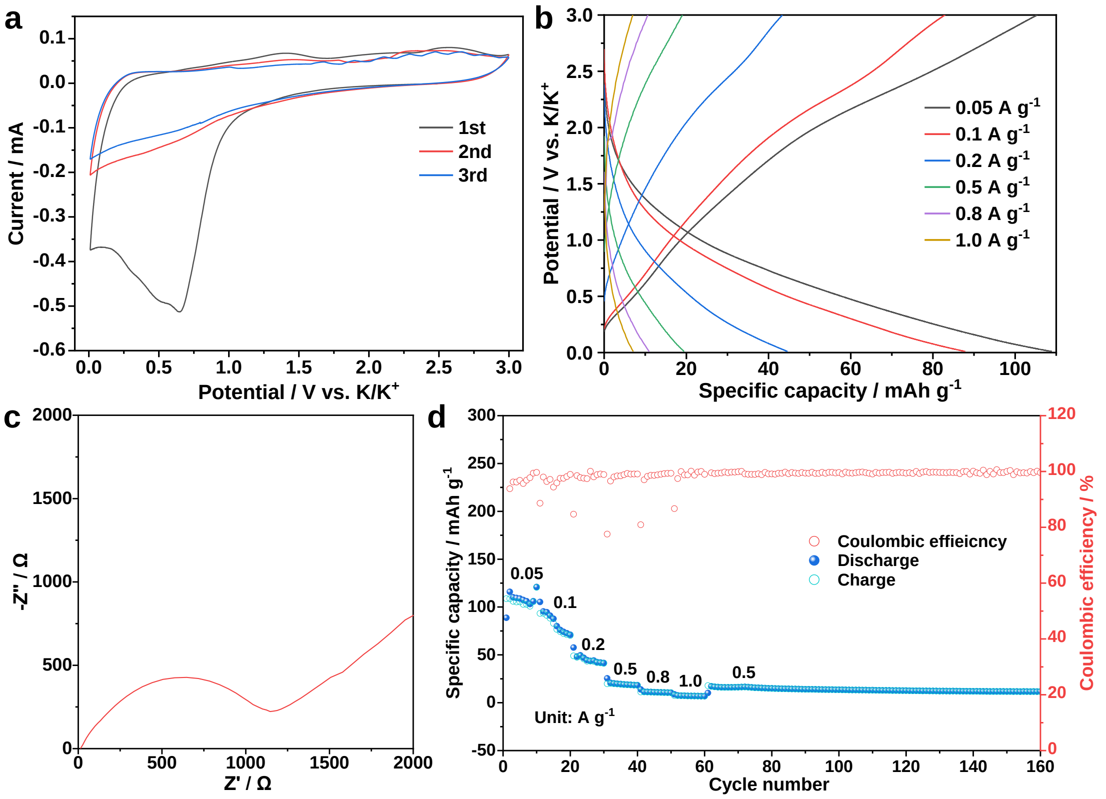

Figure S32. Electrochemical performances for K-ion battery with Co_2C materials. a) CV curves for the first three cycles of Co_2C electrode at 0.2 mV s^{-1}; b) selected charge-discharge profiles of Co_2C electrode at varied current density; c) electrochemical impedance spectra of the Co_2C electrode; and d) rate capacities and cycling performance of Co_2C electrode at varied current density.

Notes:

The specific capacities reached 105.4, 88.4 and 43.6 mAh g^{-1}, when the current densities increased from 0.05, 0.1 to 0.2 A g^{-1}, respectively, with good reversibility, cycling performance, and high coulombic efficiency.

Table S1 Structural parameters of the samples obtained from EXAFS fitting.

Sample	Bond type	N	R (Å)	ΔE_0 (eV)	$\sigma^2 \times 10^3$ (Å^2)	R-factor
Co foil	Co-Co	12.0	2.49			
	Co-Co	6.0	3.57			
Co_2C	Co-C	2.4	1.91	-12.7	4.9	0.013
	Co-Co	3.7	2.46	-11.5	5.2	
	Co-Co	1.8	2.89	-11.5	6.9	

N, coordination number; R, distance between absorber and backscatter atoms; ΔE_0, inner potential correction to account for the difference in the inner potential between the sample and reference compound. σ^2, Debye–Waller factor; S_0^2 fitting from the Pt sample was defined as 0.89.

Table S2 Performance comparison of different photocatalysts for CO_2 hydrogenation under varied conditions.

Catalyst	Temp. (°C)	H_2:CO_2	Light source	R_{CO2}[c]	CH_4 rate[d]	CO rate[e]	S_{CO}[f]	Ref.
Co_2C	200	4:1	Dark	3.7	0.2	3.5	94.6	This work
Co_2C	250	4:1	Dark	20.8	1.4	19.3	93.2	
Co_2C	300	4:1	Dark	65.3	9.1	55.6	85.2	
Nb_2C	400	2:2	Dark	0.01	-	-	-	[6]
Ni/Nb_2C	250	2:2	Dark	0.6	0.48	0.12	20.0	
β-Mo_2C	230	3:1	Dark	3.3	0.4	2.2	67.0	[7]
2D-Mo_2C	230	3:1	Dark	26.8	6.1	17.0	65.0	
Mo_2CT_x	230	3:1	Dark	3.7	1.0	1.9	54.0	
Fe_5C_2	320	3:1	Dark	40.0	18.4	1.1	2.8	[8]
Co_2C	-[a]	4:1	2.1 W cm^{-2}/300-W Xe lamp	13.9	0.5	13.4	96.3	This work
Nb_2C	-[a]	1:1[b]	1.5 W cm^{-2}/300-W Xe lamp	0.01	0.004	0.007	60.0	[6]

Ni/Nb_2C	-[a]	1:1[b]	1.5 W cm^{-2}/300-W Xe lamp	87.0	72.5	14.5	83.4	
Ti_3C_2	-[a]	1:1[b]	1.5 W cm^{-2}/300-W Xe lamp	0.02	0.004	0.013	75.0	
Fe_3C	-[a]	1:3	2.1 W cm^{-2}/300-W Xe lamp	10.9	9.9		<1.0	[9]
Pt/Al_2O_3	-[a]	4:1[b]	300-W Xe lamp	11.3	1.8	9.5	84.5	
Rh/Al_2O_3	-[a]	4:1[b]	300-W Xe lamp	165.4	164.5	0.9	0.5	[10]
Ir/Al_2O_3	-[a]	4:1[b]	300-W Xe lamp	1.4	0.9	0.5	36.7	
Co_2C	244	4:1	1.9 W cm^{-2}/420–780 nm	28.7	5.6	22.8	79.4	
Co_2C	256	4:1	1.25 W cm^{-2}/420–780 nm	29.2	4.5	24.5	83.6	This work
Co_2C	297	4:1	1.25 W cm^{-2}/420–780 nm	67.8	14.7	52.3	77.1	
$H_zIn_2O_{3-x}(OH)_y$	300	1:1	2.0 W cm^{-2}/300-W Xe lamp		-	0.43	95.0	[11]
Pd/WO_3	250	1:1	1.4 W cm^{-2}/Full	-	-	1.3	99.0	[12]
Pd/ZnO	250	3:1	500-W Hg lamp	7.4	-	3.8	52.0	[13]
Rh/TiO_2	250	3:1	1.0 W cm^{-2}/LED365	-	88		<2.0	[14]

Pt/Al_2O_3	250	6:1	0.7 W cm^{-2}/340-800 nm	7.2	-	1.92	26.7	[15]
Ru/Al_2O_3	164	5:1	0.1 W cm^{-2}/AM1.5	5.2	5.2	0	0	[16]
Ru/Al_2O_3	220	5:1	0.62 W cm^{-2}/Full	22.7	22.7	0	0	[17]

[a] Under illumination without external heat.

[b] In the batch reactors.

[c] CO_2 conversion rate, the unit is $mmol_{CO2}$ g^{-1}_{cat} h^{-1}.

[d] CH_4 production rate, the unit is $mmol_{CH4}$ g^{-1}_{cat} h^{-1}.

[e] CO production rate, the unit is $mmol_{CO}$ g^{-1}_{cat} h^{-1}.

[f] CO production selectivity (%).

References

[1] a) S. Kjeldgaard, I. Dugulan, A. Mamakhel, M. Wagemaker, B. B. Iversen, A. Bentien, *R. Soc. Open Sci.* **2021**, *8*, 201779; b) J. Su, Y. Yang, G. Xia, J. Chen, P. Jiang, Q. Chen, *Nat. Commun.* **2017**, *8*, 14969.

[2] P. Hasin, *J. Phys. Chem. C* **2014**, *118*, 4726-4732.

[3] V. G. Harris, Y. Chen, A. Yang, S. Yoon, Z. Chen, A. L. Geiler, J. Gao, C. N. Chinnasamy, L. H. Lewis, C. Vittoria, E. E. Carpenter, K. J. Carroll, R. Goswami, M. A. Willard, L. Kurihara, M. Gjoka, O. Kalogirou, *J. Phys. D: Appl. Phys.* **2010**, *43*, 165003.

[4] a) R. Zheng, C. Shu, Z. Hou, A. Hu, P. Hei, T. Yang, J. Li, R. Liang, J. Long, *ACS Appl. Mater. Interfaces* **2019**, *11*, 46696-46704; b) G. Li, N. Li, S. Peng, B. He, J. Wang, Y. Du, W. Zhang, K. Han, F. Dang, *Adv. Energy Mater.* **2020**, *11*, 2002721.

[5] H. Xu, L. Zhao, X. Liu, Q. Huang, Y. Wang, C. Hou, Y. Hou, J. Wang, F. Dang, J. Zhang, *Adv. Funct. Mater.* **2020**, *30*, 2006188.

[6] Z. Wu, C. Li, Z. Li, K. Feng, M. Cai, D. Zhang, S. Wang, M. Chu, C. Zhang, J. Shen, Z. Huang, Y. Xiao, G. A. Ozin, X. Zhang, L. He, *ACS Nano* **2021**, *15*, 5696-5705.

[7] H. Zhou, Z. Chen, E. Kountoupi, A. Tsoukalou, P. M. Abdala, P. Florian, A. Fedorov, C. R. Muller, *Nat. Commun.* **2021**, *12*, 5510.

[8] J. Liu, G. Zhang, X. Jiang, J. Wang, C. Song, X. Guo, *Catal. Today* **2021**, *371*, 162-170.

[9] C. Song, X. Liu, M. Xu, D. Masi, Y. Wang, Y. Deng, M. Zhang, X. Qin, K. Feng, J. Yan, J. Leng, Z. Wang, Y. Xu, B. Yan, S. Jin, D. Xu, Z. Yin, D. Xiao, D. Ma, *ACS Catal.* **2020**, *10*, 10364-10374.

[10] X. Meng, T. Wang, L. Liu, S. Ouyang, P. Li, H. Hu, T. Kako, H. Iwai, A. Tanaka, J. Ye, *Angew. Chem. Int. Ed.* **2014**, *126*, 11662-11666.

[11] Z. Zhang, C. Mao, D. M. Meira, P. N. Duchesne, A. A. Tountas, Z. Li, C. Qiu, S. Tang, R. Song, X. Ding, J. Sun, J. Yu, J. Y. Howe, W. Tu, L. Wang, G. A. Ozin, *Nat. Commun.* **2022**, *13*, 1512.

[12] Y. F. Li, N. Soheilnia, M. Greiner, U. Ulmer, T. Wood, A. A. Jelle, Y. Dong, A. P. Y. Wong, J. Jia, G. A. Ozin, *ACS Appl. Mater. Interfaces* **2019**, *11*, 5610-5615.

[13] D. Wu, K. Deng, B. Hu, Q. Lu, G. Liu, X. Hong, *ChemCatChem* **2019**, *11*, 1598-1601.

[14] X. Zhang, X. Q. Li, M. E. Reish, D. Zhang, N. Q. Su, Y. Gutierrez, F. Moreno, W. T. Yang, H. O. Everitt, J. Liu, *Nano Lett.* **2018**, *18*, 1714-1723.

[15] Z. Zhao, D. E. Doronkin, Y. Ye, J.-D. Grunwaldt, Z. Huang, Y. Zhou, *Chin. J. Catal.* **2020**, *41*, 286-293.

[16] F. Sastre, C. Versluis, N. Meulendijks, J. Rodriguez-Fernandez, J. Sweelssen, K. Elen, M. K. Van Bael, T. den Hartog, M. A. Verheijen, P. Buskens, *ACS Omega* **2019**, *4*, 7369-7377.

[17] R. Grote, R. Habets, J. Rohlfs, F. Sastre, N. Meulendijks, M. Xu, M. A.

Verheijen, K. Elen, A. Hardy, M. K. Van Bael, T. Hartog, P. Buskens, *ChemCatChem* **2020**, *12*, 5618-5622.
